# Tobacco Related Attitudes and Behaviours in Relation to Exposure to the Tackling Indigenous Smoking Program: Evidence from the Mayi Kuwayu Study

**DOI:** 10.3390/ijerph182010962

**Published:** 2021-10-19

**Authors:** Rubijayne Cohen, Raglan Maddox, Mikala Sedgwick, Katherine A. Thurber, Makayla-May Brinckley, Eden M. Barrett, Raymond Lovett

**Affiliations:** National Centre for Epidemiology and Population Health, Research School of Population Health, The Australian National University, Canberra, ACT 2601, Australia; Raglan.Maddox@antlineu.edu.au (R.M.); Mikala.Sedgwick@anu.edu.au (M.S.); Katherine.Thurber@anu.edu.au (K.A.T.); Makayla-May.Brinckley@anu.edu.au (M.-M.B.); Eden.Barrett@anu.edu.au (E.M.B.); Raymond.Lovett@anu.edu.au (R.L.)

**Keywords:** indigenous peoples, Aboriginal, Torres Strait Islander, tobacco, smoking, Tackling Indigenous Smoking, smoking intervention, cessation, Australia

## Abstract

Smoking is the leading contributor to the burden of disease and mortality for Aboriginal and Torres Strait Islander peoples, with an estimated 37% of all Aboriginal and Torres Strait Islander deaths attributed to smoking. The Tackling Indigenous Smoking (TIS) program was implemented to support people to quit smoking, prevent initiation, and reduce exposure to second-hand smoke. Analysis of baseline (2018–2020) data from a large-scale cohort study was conducted to quantify smoking-related attitudes and behaviours among Aboriginal and Torres Strait Islander adults, overall and in relation to exposure to the TIS program. Most results were similar for TIS and non-TIS, but there was a significantly lower prevalence of smoking inside households (PR0.85; 95% CI: 0.74, 0.97), smoking ≥21 cigarettes per day (PR0.79; 95% CI: 0.62, <1.00), and smoking a first cigarette within 5 min of waking (PR0.87; 95% CI: 0.76, <1.00) in TIS-funded compared to non-TIS-funded areas. Findings from the analysis highlight encouraging anti-smoking attitudes and behaviours across TIS-funded and non-TIS-funded areas, and serve as a basis for future analysis of change in outcomes over time associated with exposure to a large multi-mode population health program (TIS).

## 1. Introduction

Tobacco products were introduced to Australia through colonisation and were used to establish relationships with Aboriginal peoples and later Torres Strait Islander peoples. Tobacco was systematically used in government-issued rations and as payment for labour until as late as 1968 [1]. These systemic practices promoted, embedded and normalised widespread tobacco use within the Aboriginal and Torres Strait Islander population. As a result, smoking is the leading contributor to the burden of disease and mortality for Aboriginal and Torres Strait Islander peoples [2], with an estimated 37% of all Aboriginal and Torres Strait Islander deaths attributed to smoking [3]. The Aboriginal and Torres Strait Islander tobacco epidemic is unique due to the extended duration of high prevalence, similarity in prevalence among men and women, and distinct trends between urban, regional and remote settings [4].

While tobacco’s contribution to the burden of disease and mortality is substantial, recent analysis of Aboriginal and Torres Strait Islander adult smoking prevalence (≥18 years) shows an absolute decline of 9.8% over the past 15 years (from 50.0% in 2004/05 to 40.2% in 2018/19). This equates to almost 50,000 fewer Aboriginal and Torres Strait Islander adult daily smokers than there would have been if smoking prevalence had remained at 2004/2005 levels [5].

Communities, health services and governments have developed and implemented a range of programs and policies to support people to quit smoking, to prevent initiation, and to reduce exposure to second-hand smoke. For example, national, state and territory tobacco control programs and policies include annual tax excise increases, mass media communication campaigns, smoke-free legislations and access to Quitlines to support cessation. In addition, Aboriginal Community Controlled Health Organisations (ACCHOs) provide healthcare to Aboriginal and Torres Strait Islander peoples across the country, including providing information and support for smoking cessation [6]. These efforts are likely to have contributed to observed reductions in tobacco use among the Aboriginal and Torres Strait Islander population across the country. The Tackling Indigenous Smoking (TIS) program is a national initiative that takes a multipronged population approach. It is the first large-scale, long-term, comprehensive public health program that aims to reduce smoking among the Aboriginal and Torres Strait Islander population. Initiated in 2010, TIS encourages partnerships and collaboration across services to promote smoke-free attitudes and behaviours [6]. TIS provides Regional Tobacco Grants to 37 organisations, predominantly ACCHOs. These grants allow local areas to determine and implement activities and programs suited to their community context and needs [1]. In doing so, TIS has considerable diversity and variation in implementation. Across the country, TIS has incorporated a mix of the following activities over time:

- Smoke-free spaces;
- Brief interventions—including but not limited to screening, assessment and brief advice in relation to tobacco use;
- Community engagement, education and training;
- Media campaigns and social media campaigns;
- One-on-one or group smoking cessation support;
- Promotional resources—posters and pamphlets;
- Provision of medicines to help people stop smoking, such as Nicotine Replacement Therapy (NRT), varenicline, and bupropion (no longer funded) and
- Anti-e-cigarette/anti-vaping activities.

Further detail and information about the TIS program can be found elsewhere [1,6,7].

Assessing the impact of multifaceted interventions, such as TIS (a multi-site, multi-intervention program), at the population level is complex. Often, population health and health service interventions are incompatible with evaluation designs, which randomly assign individuals or locations to intervention or control groups; use of these experimental designs are often inappropriate and/or unethical [7]. Cohort studies present a non-experimental alternative evaluation design, since they can contain exposed and unexposed individuals or locations, and can be large-scale and responsive to policy timing [8]. Such features are ideal to provide insight into the long-term effects of population-level interventions and policies in a real-world setting. Cross-sectional analysis of baseline data from such a cohort study can provide insight into intended outcomes and their association with program exposure, at one time-point. Where data are available, longitudinal analysis of change in outcomes in relation to intervention exposure can provide insight into the impacts of programs.

This paper aims to quantify smoking-related attitudes and behaviours among Aboriginal and Torres Strait Islander adults, and relationships with exposure to a large-scale population-based tobacco control program (TIS) targeted at the Aboriginal and Torres Strait Islander population in Australia, using baseline data from a large-scale cohort study. It is intended to provide a snapshot of tobacco-related outcomes at one time-point and their association with intervention (TIS) exposure, serving as the foundation for future longitudinal analysis when follow-up data become available.

## 2. Materials and Methods

### 2.1. Mayi Kuwayu Study

Mayi Kuwayu: The National Study of Aboriginal and Torres Strait Islander Wellbeing (the Mayi Kuwayu Study) is the largest existing prospective cohort study of Aboriginal and Torres Strait Islander peoples in Australia. Participants were recruited via mixed methods, including postal questionnaire (identified through the Medicare Enrolment Database), in-community recruitment, community partners, or online questionnaire, from October 2018. All Mayi Kuwayu Study data included in the current analysis were based on self-reported responses, with the exception of remoteness, which was derived based on postcode. Details of the study design are provided elsewhere [9,10].

The current study is a cross-sectional analysis of Aboriginal and Torres Strait Islander people ≥ 16 years who participated in the Mayi Kuwayu Study and whose data are included in Release 2.0 (*N* = 9485). This includes all survey responses scanned and received by 1 May 2020 and participants from all modes of survey completion (mail out, online, face-to-face). Participants were excluded from the analysis if they had missing data on age, gender or smoking status (*n* = 936, for a final sample of *N* = 8549).

### 2.2. Governance, Ethics and the Research Team

The Mayi Kuwayu Study arose from community-identified priorities and was developed over four years in consultation with Aboriginal and Torres Strait Islander peoples across Australia. The study is overseen by an Aboriginal and Torres Strait Islander governance group consisting of peak Aboriginal and Torres Strait Islander health organisations. In addition, all proposed data use is assessed according to Indigenous Data Sovereignty principles and approved by an independent Indigenous Data Governance committee, known as the Mayi Kuwayu Data Governance Committee (MKDGC). This research was approved by the MKDGC (including the manuscript) and conducted with ethics approvals from relevant Aboriginal and Torres Strait Islander organisations, and from national, state and territory Human Research Ethics Committees (HRECs).

We recognise that the research team members’ worldviews can influence our perspectives and values, including how the study was conducted and findings interpreted. Our team brings Aboriginal lived experience (R.C., M.S., M.-M.B., R.L.) and experience in public health and epidemiology and decolonizing methodologies (all authors). Aboriginal and Torres Strait Islander people were involved at all stages of the research for both the Mayi Kuwayu Study and the current project. Project conception, design, data collection, analysis and interpretation were conducted by, with and for the benefit of Aboriginal and Torres Strait Islander people.

### 2.3. Outcomes: Smoking-Related Attitudes and Behaviours

We examined a range of smoking-related attitudes and smoking-related behaviours as outlined in Table 1.

### 2.4. Main Exposure: TIS-Funded Areas

Key informant consultations and Australian Government Department of Health documentation were used to define TIS geographic boundaries; this was supplemented by information from the Department of Health Tackling Indigenous Smoking Services map where required [11]. Participants were classified as residing in either a TIS-funded area or non-TIS-funded area (“TIS” or “non-TIS”) based on their postcode; for this analysis, we did not have access to finer level geographic data on participants’ location. The geographic boundaries of TIS service provision do not neatly align with postcode boundaries in all cases. Therefore, a conservative approach was adopted, with postcodes only coded as TIS if their entire area was within a TIS service boundary (i.e., 100% population coverage). All partially serviced postcodes were coded as non-TIS. This conservative approach was chosen to maximize the specificity of areas coded as TIS.

### 2.5. Demographic Variables

Demographic variables included age, gender, highest level of education, remoteness (ASGS) and Aboriginal and/or Torres Strait Islander identity.

### 2.6. Statistical Analysis

Descriptive analysis of participant demographic variables is presented as the percentage and number, overall and by TIS exposure (Table 2). We then present a descriptive overview of smoking-related attitudes (Table 3) and behaviours (Table 4), overall and by TIS/non-TIS. These smoking-related outcomes are presented by categories of age, gender, and remoteness in Supplementary Tables S1–S6.

Log binomial regression models were used to calculate the prevalence ratio (PR) and 95% Confidence Interval (CI) of each smoking-related attitude and behaviour (coded as binary variables; see Supplementary Tables S7–S8 for details) in relation to TIS/non-TIS; analyses were conducted for all participants, currents smokers, or past smokers, as appropriate. Participants missing data on the outcome of interest are excluded from that model. Associations were run (1) unadjusted (crude), (2) adjusted for broad age groups (16–35, 36–55 and ≥56 years) and gender (men and women); the latter results are presented in the text. As this analysis was exploratory in nature, we adopted this minimally adjusted approach. A sensitivity analysis additionally adjusted for broad remoteness category (major cities; inner regional; outer regional, remote and very remote) as it was considered conceptually to be an additional potential confounder of the exposure–outcome associations (see Supplementary Tables S9–S11).

For all descriptive results, cells *n* ≤ 5 were suppressed (with the exception of the missing category) to protect confidentiality. Analyses were conducted using Stata 16.

## 3. Results

### 3.1. Participant Characteristics

Participants included 8549 Aboriginal and/or Torres Strait Islander adults aged ≥16 years. Over 40 percent (41.7%) of participants were classified as living in TIS-funded areas; the remaining 58.3% were classified as living in non-TIS-funded areas. Sixty percent of participants (61.5%) were women, 42.1% were from major cities and 42.2% were aged ≥55 (Table 2). Age, gender, education and Indigeneity profiles were similar across TIS and non-TIS as detailed in Table 2. A lower percentage of TIS compared to non-TIS participants resided in major cities (33.2% versus 48.5%) and very remote areas (3.9% versus 8.2%), but a higher percentage resided in inner regional areas (35.7% versus 23.7%) and remote areas (5.9% versus 1.2%).

**Table 2:** Demographic characteristics of Mayi Kuwayu Study baseline participants, overall and by TIS/non-TIS.

	Total Sample % (*n*)	TIS % (*n*)	Non-TIS % (*n*)
Overall	*N* = 8549	*N* = 3567	*N* = 4982
Age group (years)			
16–24	10.6 (902)	10.6 (377)	10.5 (525)
25–34	13.3 (1136)	14.0 (499)	12.8 (637)
35–44	14.8 (1268)	14.9 (532)	14.8 (736)
45–54	19.1 (1637)	18.5 (659)	19.6 (978)
≥55	42.2 (3606)	42.1 (1500)	42.3 (2106)
Gender			
Men	≤38.4 (≤3290)	≤37.8 (≤1350)	≤38.9 (≤1940)
Women	61.5 (5255)	62.1 (2214)	61.0 (3041)
Do not identify as a man or a woman	≤0.1 (≤10)	≤0.1 (≤5)	≤0.1 (≤5)
Education			
Year 10 or below	43.9 (3750)	44.0 (1570)	43.8 (2180)
Year 12 or trade certificate	37.5 (3203)	38.2 (1363)	36.9 (1840)
University qualification/s	17.4 (1491)	16.7 (597)	17.9 (894)
Missing	1.2 (105)	1.0 (37)	1.4 (68)
Level of remoteness			
Major city	42.1 (3600)	33.2 (1184)	48.5 (2416)
Inner regional	28.7 (2452)	35.7 (1272)	23.7 (1180)
Outer regional	18.5 (1579)	21.2 (757)	16.5 (822)
Remote	3.2 (270)	5.9 (210)	1.2 (60)
Very remote	6.4 (546)	3.9 (138)	8.2 (408)
Missing	1.2 (102)	0.2 (6)	1.9 (96)
Indigeneity			
Aboriginal	93.1 (7961)	92.2 (3290)	93.8 (4671)
Torres Strait Islander	3.1 (261)	3.5 (124)	2.7 (137)
Both Aboriginal and Torres Strait Islander	3.8 (327)	4.3 (153)	3.5 (174)

### 3.2. Smoking-Related Attitudes

Overall, 59.0% of participants did not believe that non-smokers miss out on gossip or yarning and 60.0% reported community disapproval of smoking (Table 3). Personal health, cost, and health of family were the most commonly reported reasons for current smokers wanting to quit and for past smokers quitting. Among current smokers, 76.3% reported wanting to quit.

**Table 3:** Smoking-related attitudes in the total sample, current smokers only, and past smokers only, overall and by TIS/non-TIS.

**Total Sample**			
	**Overall *N* = 8549** **% (*n*)**	**TIS *N* = 3567** **% (*n*)**	**Non-TIS *N* = 4982** **% (*n*)**
Do you agree that non-smokers miss out on gossip or yarning?			
Not at all	59.0 (5045)	60.0 (2140)	58.3 (2905)
A little bit	16.7 (1427)	16.8 (598)	16.6 (829)
A fair bit/A lot	15.0 (1285)	14.7 (523)	15.3 (762)
Missing	9.3 (792)	8.6 (306)	9.8 (486)
Do you agree that your community disapproves of smoking?			
Not at all	27.8 (2374)	27.2 (972)	28.1 (1402)
A little bit	23.9 (2044)	24.9 (887)	23.2 (1157)
A fair bit/A lot	36.1 (3089)	36.1 (1288)	36.2 (1801)
Missing	12.2 (1042)	11.8 (420)	12.5 (622)
Do you agree that smoking is not that risky?			
Not at all	49.3 (4218)	49.4 (1763)	49.3 (2455)
A little bit	7.0 (597)	6.7 (240)	7.2 (357)
A fair bit/A lot	31.7 (2708)	32.2 (1150)	31.3 (1558)
Missing	12.0 (1026)	11.6 (414)	12.3 (612)
**Current smokers**			
	**Overall *N* = 2216** **% (*n*)**	**TIS *N* = 931** **% (*n*)**	**Non-TIS *N* = 1285** **% (*n*)**
Do you think your smoking has made you sick?			
No	36.8 (816)	37.8 (352)	36.1 (464)
Yes	34.9 (773)	35.7 (332)	34.3 (441)
Unsure	25.9 (575)	24.2 (225)	27.2 (350)
Missing	2.3 (52)	2.4 (22)	2.3 (30)
Do you think your smoking will make you sick in the future?			
Not at all	8.4 (186)	7.2 (67)	9.3 (119)
A little bit	20.2 (448)	21.5 (200)	19.3 (248)
A fair bit/A lot	55.6 (1232)	56.3 (524)	55.1 (708)
Unsure	14.0 (310)	12.9 (120)	14.8 (190)
Missing	1.8 (40)	2.1 (20)	1.6 (20)
Do you want to quit smoking?			
Not at all	10.7 (238)	10.4 (97)	11.0 (141)
A little bit	21.8 (482)	20.6 (192)	22.6 (290)
A fair bit/A lot	54.5 (1207)	56.1 (522)	53.3 (685)
Unsure	10.8 (240)	10.5 (98)	11.1 (142)
Missing	2.2 (49)	2.4 (22)	2.1 (27)
Why do you want to quit? *			
Advertising against smoking	3.2 (71)	2.5 (23)	3.7 (48)
Medical advice	17.2 (382)	17.2 (160)	17.3 (222)
My Health	55.5 (1230)	56.0 (521)	55.2 (709)
Health of my family	24.1 (533)	24.9 (232)	23.4 (301)
Cost	48.1 (1065)	48.3 (450)	47.9 (615)
Pregnancy	0.9 (19)	1.2 (11)	0.6 (8)
Too many non-smoking areas	4.5 (99)	4.3 (40)	4.6 (59)
Pressure from family or friends	14.6 (323)	14.2 (132)	14.9 (191)
Other	6.3 (139)	6.2 (58)	6.3 (81)
Missing or indicated does not want to quit	15.6 (345)	14.9 (139)	16.0 (206)
**Past smokers**			
	**Overall *N* = 2841** **% (*n*)**	**TIS *N* = 1209** **% (*n*)**	**Non-TIS *N* = 1632** **% (*n*)**
Do you think your past smoking has made you sick?			
No	44.9 (1277)	44.7 (541)	45.1 (736)
Yes	24.3 (690)	24.5 (296)	24.1 (394)
Unsure	23.1 (655)	22.9 (277)	23.2 (378)
Missing	7.7 (219)	7.9 (95)	7.6 (124)
Do you think your past smoking will make you sick in the future?			
Not at all	27.2 (773)	26.7 (323)	27.6 (450)
A little bit	20.7 (587)	21.3 (258)	20.2 (329)
A fair bit/A lot	12.8 (363)	13.2 (160)	12.4 (203)
Unsure	32.5 (922)	31.5 (381)	33.1 (541)
Missing	6.9 (196)	7.2 (87)	6.7 (109)

* Indicates a question where multiple responses are possible. This means that the column percentages may sum to more than 100%.

### 3.3. Smoking Behaviours

Overall, 40.8% of Mayi Kuwayu Study participants were never smokers, 25.9% were current smokers and 33.2% were past smokers, with smoking status similar across TIS and non-TIS (Table 4). Forty-five percent (44.9%) of current smokers reported trying to quit and 48.4% reported trying to reduce smoking in the past year. Twelve percent (11.5%) of current smokers and 5.8% of past smokers reported ever participating in a quit smoking program, service or activity, with similar percentages across TIS and non-TIS. Among past smokers, 47.6% reported initiating smoking when they were <15 years. The majority of past smokers quit more than five years ago (66.8%).

**Table 4:** Smoking-related behaviours in the total sample, current smokers, and past smokers, overall and by TIS/non-TIS.

**Total Sample**			
	**Overall *N* = 8549** **% (*n*)**	**TIS *N* = 3567** **% (*n*)**	**Non-TIS *N* = 4982** **% (*n*)**
Smoking status			
Never smoker	40.8 (3492)	40.0 (1427)	41.4 (2065)
Current smoker	25.9 (2216)	26.1 (931)	25.8 (1285)
Past smoker	33.2 (2841)	33.9 (1209)	32.8 (1632)
Does anyone smoke in your home or in your car? *			
No smoking inside or outside the home, or in the car	68.4 (5845)	68.9 (2458)	68.0 (3387)
Inside the home	9.4 (800)	8.4 (301)	10.0 (499)
Outside the home	19.4 (1659)	19.3 (689)	19.5 (970)
In the car	8.4 (719)	8.0 (284)	8.7 (435)
Missing	4.4 (379)	4.3 (155)	4.5 (224)
**Current smokers**			
	**Overall *N* = 2216** **% (*n*)**	**TIS *N* = 931** **% (*n*)**	**Non-TIS *N* = 1285** **% (*n*)**
How old were you when you started smoking?			
1–15 years old	53.9 (1195)	54.1 (504)	53.8 (691)
16–18 years old	28.7 (637)	29.4 (274)	28.2 (363)
19–24 years old	8.9 (197)	8.1 (75)	9.5 (122)
25–34 years old	3.4 (75)	2.7 (25)	3.9 (50)
≥35 years old	1.9 (42)	2.6 (24)	1.4 (18)
Missing	3.2 (70)	3.1 (29)	3.2 (41)
In the past year, have you tried to quit or reduce the amount you smoke? *			
Tried to quit smoking	44.9 (994)	45.5 (424)	44.4 (570)
Tried to reduce smoking	48.4 (1073)	49.0 (456)	48.0 (617)
Have not tried to quit or reduce smoking	18.1 (400)	17.2 (160)	18.7 (240)
Missing	2.8 (62)	2.4 (22)	3.1 (40)
How often do you smoke?			
Less than weekly	5.2 (115)	4.8 (45)	5.4 (70)
Weekly (not every day)	11.4 (252)	10.5 (98)	12.0 (154)
Every day	81.5 (1806)	83.1 (774)	80.3 (1032)
Missing	1.9 (43)	1.5 (14)	2.3 (29)
How many cigarettes do you usually smoke in one day?			
1–10 cigarettes per day	53.1 (1177)	55.2 (514)	51.6 (663)
11–20 cigarettes per day	30.3 (671)	30.1 (280)	30.4 (391)
21–30 cigarettes per day	8.9 (198)	7.1 (66)	10.3 (132)
≥31 cigarettes per day	2.3 (50)	2.3 (21)	2.3 (29)
Missing or smokes less than daily	5.4 (120)	5.4 (50)	5.4 (70)
How soon after waking do you usually have your first cigarette?			
5 min or less	26.6 (589)	24.5 (228)	28.1 (361)
6–30 min	36.6 (810)	37.9 (353)	35.6 (457)
31–60 min	16.1 (356)	17.1 (159)	15.3 (197)
61 or more minutes	9.3 (205)	10.0 (93)	8.7 (112)
Don’t smoke every day	8.4 (187)	7.9 (74)	8.8 (113)
Missing	3.1 (69)	2.6 (24)	3.5 (45)
Have you ever participated in any quit smoking program, service or activity?			
Not selected	88.5 (1962)	87.6 (816)	89.2 (1146)
Yes	11.5 (254)	12.4 (115)	10.8 (139)
**Past smokers**			
	**Overall *N* = 2841** **% (*n*)**	**TIS *N* = 1209** **% (*n*)**	**Non-TIS *N* = 1632** **% (*n*)**
How old were you when you started smoking?			
1–15 years old	47.6 (1351)	46.2 (558)	48.6 (793)
16–18 years old	29.7 (845)	30.7 (371)	29.0 (474)
19–24 years old	9.7 (276)	9.4 (114)	9.9 (162)
25–34 years old	3.3 (95)	4.0 (48)	2.9 (47)
≥35 years old	1.2 (34)	1.0 (12)	1.3 (22)
Missing	8.4 (240)	8.8 (106)	8.2 (134)
When you used to smoke, how often did you smoke?			
Less than weekly	6.8 (194)	6.3 (76)	7.2 (118)
Weekly (not every day)	11.2 (317)	11.5 (139)	10.9 (178)
Every day	75.2 (2136)	74.7 (903)	75.6 (1233)
Missing	6.8 (194)	7.5 (91)	6.3 (103)
When you used to smoke, how many cigarettes did you usually smoke in one day?			
1–10 cigarettes per day	39.8 (1130)	41.3 (499)	38.7 (631)
11–20 cigarettes per day	27.0 (768)	26.3 (318)	27.6 (450)
21–30 cigarettes per day	15.6 (443)	15.6 (188)	15.6 (255)
≥31 cigarettes per day	8.8 (250)	7.9 (95)	9.5 (155)
Missing or less than daily	8.8 (250)	9.0 (109)	8.6 (141)
How soon after waking did you usually have your first cigarette?			
5 min or less	23.3 (662)	22.7 (274)	23.8 (388)
6–30 min	26.8 (761)	27.5 (333)	26.2 (428)
31–60 min	14.0 (399)	13.6 (164)	14.4 (235)
61 or more minutes	13.0 (370)	13.7 (166)	12.5 (204)
Don’t smoke every day	13.8 (393)	12.7 (154)	14.6 (239)
Missing	9.0 (256)	9.8 (118)	8.5 (138)
Have you ever participated in any quit smoking program, service or activity?			
Not selected	94.2 (2675)	94.1 (1138)	94.2 (1537)
Yes	5.8 (166)	5.9 (71)	5.8 (95)
How long ago did you quit?			
1 to 5 months	5.6 (160)	5.9 (71)	5.5 (89)
6 months to a year	3.5 (99)	3.9 (47)	3.2 (52)
1 to 2 years	6.0 (171)	6.3 (76)	5.8 (95)
2 to 5 years	10.4 (296)	10.3 (124)	10.5 (172)
More than 5 years	66.8 (1898)	65.5 (792)	67.8 (1106)
Missing	7.6 (217)	8.2 (99)	7.2 (118)
What led you to quit? *			
Advertising against smoking	6.2 (176)	7.0 (85)	5.6 (91)
Medical advice	15.5 (441)	15.7 (190)	15.4 (251)
My Health	46.5 (1321)	46.9 (567)	46.2 (754)
Health of my family	18.0 (511)	17.7 (214)	18.2 (297)
Cost	28.4 (807)	28.5 (345)	28.3 (462)
Pregnancy	8.4 (239)	7.0 (85)	9.4 (154)
Too many non-smoking areas	1.8 (51)	2.1 (25)	1.6 (26)
Pressure from family or friends	11.9 (338)	12.8 (155)	11.2 (183)
Other	20.1 (572)	20.1 (243)	20.2 (329)
Missing	7.5 (212)	7.9 (96)	7.1 (116)
What helped you quit? *			
Smoking program	2.9 (83)	2.5 (30)	3.2 (53)
Quitline	1.5 (44)	1.0 (12)	2.0 (32)
Online support	≤0.3 (≤10)	--	0.4 (6)
Health professional	4.1 (117)	3.4 (41)	4.7 (76)
Family or friends	9.0 (257)	9.3 (113)	8.8 (144)
Patches, gum, inhaler (NRT)	9.4 (266)	9.6 (116)	9.2 (150)
Stop smoking medication	7.4 (209)	6.3 (76)	8.1 (133)
Quit on my own	65.8 (1870)	66.7 (806)	65.2 (1064)
Other	12.5 (354)	13.2 (159)	11.9 (195)
Missing	--	--	--

* Indicates a question where multiple responses are possible. This means that the column percentages may sum to more than 100%. -- indicates a suppressed cell due to small numbers.

### 3.4. Associations between TIS and Smoking-Related Attitudes and Behaviours

In the total sample, a significant, 15% lower prevalence of smoking inside the home was observed among TIS compared to non-TIS (PR0.85; 95% CI: 0.74, 0.97) (Table 5). There were no strong or significant associations between TIS and the other anti-smoking attitudes and behaviours examined in the total sample.

Among past smokers, a significantly lower percentage of TIS versus non-TIS participants reported wanting to quit due to pregnancy (PR0.74, 95% CI: 0.58, 0.94) (Table 6).

Among current smokers, TIS was associated with an 18% lower prevalence of anyone smoking in the home (PR0.82, 95% CI: 0.70, 0.95) compared to non-TIS (Table 7). There was also significantly lower prevalence of smoking ≥21 cigarettes per day (PR0.79; 95% CI: 0.62, <1.00), and smoking first cigarette within 5 min of waking (PR0.87; 95% CI: 0.76, <1.00) in TIS compared to non-TIS. There were not strong or significant associations between TIS and other smoking behaviours among current smokers, or for any smoking behaviours among past smokers.

## 4. Discussion

In this cohort of Aboriginal and Torres Strait Islander adults, we commonly observed anti-smoking attitudes and behaviours among current smokers, and recognition of the potential health harms of smoking among current and past smokers. Three quarters (76.2%) of current smokers reported wanting to quit, with their own health, cost, and family health common reasons reported for wanting to quit. Nearly half of all current smokers reported trying to quit in the past year (44.9%), and nearly half (48.4%) reported trying to reduce smoking, with similar patterns across TIS and non-TIS areas. However, reported participation in quit smoking programs and activities was low and not materially different between TIS and non-TIS participants. Less than 6% of all past smokers reported use of a quit smoking program or service. Previous research has indicated that the majority of Aboriginal and Torres Strait Islander past smokers quit unaided [12], which may partially explain these results. Further, participants in TIS areas may have been exposed to TIS activities (e.g., media campaign or promotional resources) without actively participating in a quit-smoking activity. In addition, two-thirds of past smokers in this cohort had quit smoking more than 5 years ago and, therefore, their quitting may have occurred prior to TIS implementation.

The analysis shows a generally similar profile of anti-smoking attitudes and behaviours in TIS and non-TIS areas in 2018–2020, with a few key exceptions. While the observed magnitude of associations in this study was small in most cases, the observed effect sizes are consistent with the effect sizes plausibly expected for a population level, multi component intervention [13]. What might be perceived as small effects at the individual level can be substantial at the population level, especially in the context of a population health intervention with relatively broad population reach, such as TIS [13]. Even small effect sizes can be both important and actionable to improve population health. Further, the TIS intervention comprises a combination of components of varied intensity (i.e., ranging from tailored one-on-one or small-group support, to lower-touch components such as media campaigns), with reach varying between activities. Effect sizes should be interpreted accordingly.

In the total sample, residing in a TIS-funded area was associated with a significant, 15% lower prevalence of smoking inside the home. Similarly, among current smokers, residing in a TIS-funded area was also significantly associated with an 18% lower prevalence of smoking inside the home. Previous research on smoke-free homes and workplaces has demonstrated links between having a smoke-free home and other anti-smoking attitudes and behaviours, such as wanting to quit, having made a quit attempt in the past year, and having ever stayed quit for a month or more [12]. Therefore, the observed, lower prevalence of smoking inside the home among all participants and among current smokers residing in TIS versus non-TIS areas is a promising finding for subsequent smoking behaviours among those participants.

For current smokers, residing in a TIS-funded area compared to a non-TIS-funded area was also associated with a significant 21% lower prevalence of high smoking intensity (≥21 cigarettes per day). Given that increasing smoking intensity is associated with increasing mortality risk for Aboriginal and Torres Strait Islander adults [3], the observed lower smoking intensity within TIS-funded areas is likely to reduce mortality risk for these participants. Smoking intensity is also an indicator of nicotine dependence. We also observed a 13% lower prevalence of another key indicator of nicotine dependence (smoking a first cigarette within 5 min of waking [14]) among current smokers in TIS compared to non-TIS areas. Even after accounting for smoking intensity, time until first cigarette is a strong predictor of smoking cessation as well as health outcomes [15]. These findings are likely to be interrelated and/or reinforcing; for example, research from other populations has shown that smoke-free homes are associated with reduced smoking intensity and urges to smoke in the morning and, in turn, linked to increased time to first cigarette [15,16]. It is important to note that regardless of indicators of nicotine dependence, there is evidence that Aboriginal and Torres Strait Islander peoples can quit and sustain quit attempts at any time [14]. Most past smokers in this cohort quit unaided (65.8%), consistent with the previous literature [12].

These findings are the first to explore observed differences in TIS and non-TIS areas regarding smoking-related attitudes, suggesting that most current smokers are aware of the immediate and long-term health effects of smoking. Among current smokers, it was more common in TIS compared to non-TIS funded areas to have the belief that smoking would make them sick in the future, an indication of higher awareness of the harms of smoking. Across TIS and non-TIS settings, over half of current smokers reported their health as a reason for wanting to quit—this was also the most commonly reported reason for wanting to quit. This indicates that beliefs and awareness of the potential harms of smoking are not enough to immediately change smoking behaviour among current smokers. However, these precursory attitudes could be a step on the pathway to bring about changes in smoking behaviour. The findings presented, in addition to existing evidence [1,17], suggest further positive outcomes for smoking behaviours could be forthcoming, with anti-smoking attitudes more likely to lead to a quit attempt, and to quit successfully (stay-quit).

For past smokers, residing in a TIS-funded area was significantly associated with a lower prevalence of reporting wanting to quit due to pregnancy. This finding should not be over-interpreted due to the small absolute number of participants with the outcome (e.g., 7.0% of past smokers in TIS reported wanting to quit due to pregnancy compared to 9.4% in non-TIS).

These findings provide a useful understanding into anti-smoking attitudes and behaviours among the Aboriginal and Torres Strait Islander adult population overall, and in relation to exposure to the TIS program—the first large-scale, long-term comprehensive public health programs aimed to address smoking in the population. It provides valuable insight into the current (2018–2020) state of smoking-related attitudes and behaviours in a large sample of Aboriginal and Torres Strait Islander adults, and their relationship with exposure to the TIS program. Further evaluation evidence is required to better understand what works and these findings will form a baseline for examining changes in attitudes and behaviours between the first and second wave of data collection for the Mayi Kuwayu Study.

Smoking is a complex behaviour influenced by numerous and interrelated factors. An individual’s anti-smoking attitudes, their social and cultural contexts as well as their broader environment can all influence smoking behaviour and the relative impact of these factors can fluctuate throughout the lifetime [18,19,20]. Consistent with the Theory of Triadic Influence and other models for understanding the interrelated and dynamic influences on smoking behaviours, intervening on these factors may promote an environment to prevent smoking initiation, increase successful quit attempts, and prevent relapse [16]. Anti-smoking attitudes and the normalization of smoke-free behaviours are important steps to decrease smoking prevalence. Substantial ongoing declines in Aboriginal and Torres Strait Islander smoking prevalence have been observed since 2004–2005 [5] and there is an opportunity to accelerate rates of smoking cessation and non-initiation.

### Strengths and Limitations

The Indigenous governance of, and meaningful engagement of, Aboriginal and Torres Strait Islander peoples throughout this research process is a strength of the research, and consistent with the World Health Organization’s Framework Convention on Tobacco Control (FCTC). Australia is a party to the FCTC, which highlights the need for Aboriginal and Torres Strait Islander peoples and communities to be engaged in the development, implementation and evaluation of tobacco control programs.

Another strength of the study is the large, diverse, national sample. While prevalence estimates from this study are not generalizable to the total Aboriginal and Torres Strait Islander population, findings of exposure–outcome associations are generally understood to be generalizable beyond the study sample. For example, the prevalence of smoking in this cohort is lower than is observed in the total population, consistent with the “healthy cohort” effect. Further, the age distribution in this sample is markedly older than that of the total population, with over 40% of the sample aged 55 years and older. The inclusion of these older participants is a strength of the study, and older participants are similarly represented in the TIS and non-TIS groups; variation in smoking-related outcomes by age group is provided in Supplementary Tables S2, S4 and S6.

In this study, models were minimally adjusted (by age and gender) given the exploratory nature of the study; although there was variation in many smoking-related outcomes by remoteness (Supplementary Tables S1, S3 and S5), further adjustment by remoteness did not materially alter findings (Supplementary Tables S9–S11). In addition, anti-smoking attitudes and behaviours in TIS and non-TIS areas may be shaped by a range of local and national tobacco control efforts, which we were unable to account for in our analysis.

A limitation of this research is the cross-sectional nature of the analysis, which precludes our ability to generate evidence on TIS program impact. The findings are not intended to and should not be interpreted as providing evidence of changes in smoking-related outcomes in relation to the TIS program intervention, particularly given that we lack data on outcomes pre-program implementation and were unable to analyse change in outcomes over time. It is plausible that the sites that opted to be part of the TIS program did so because they already had prevalent anti-smoking behaviours, and these pre-intervention differences may explain the observed differences at the time-point under study. However, it is also possible that the sites that opted to be part of the TIS program did so due to concern over high prevalence of smoking behaviours and low prevalence of anti-smoking behaviours. While it is not possible to accurately retrospectively capture smoking-related outcomes in this cohort at the time of program implementation, the ongoing collection of follow-up data will enable quantification of changes in outcomes during a period of program implementation, providing some insight into program impact.

We adopted a conservative approach of classifying only fully serviced postcodes (100% population coverage) as TIS, which results in the potential misclassification of TIS-exposed participants as non-TIS; this may dilute findings of exposure–outcome associations. Without finer grain geographical data (unavailable at the time of the analysis), some misclassification is inevitable and any classification would result in some extent of misclassification. In addition, the current geographic classification reflects the areas that were funded to deliver the TIS program, but does not reflect the reach or intensity of program exposure across the geographic area. To address this limitation in future research, a TIS Program Intensity Tool (a semi-quantitative survey) has been developed and administered to TIS teams nationally to better understand and quantify implementation of the TIS program.

Further work is required to accelerate reductions in tobacco use and tobacco-related morbidity and mortality among the Aboriginal and Torres Strait Islander population, across TIS and non-TIS areas. While this appears to be a priority in the draft National Preventive Health Strategy and the imminent new Implementation Plan for the National Aboriginal and Torres Strait Islander Health Plan, such strategies need to be adequately resourced, supported and informed by the strongest available evidence to urgently address the tobacco epidemic. Adequate and sustained resourcing would be expected to accelerate improvements in smoking cessation and non-uptake, leading to improved health outcomes. It would also support more equitable access to public health, health promotion and cessation programs and policies for more Aboriginal and Torres Strait Islander peoples across this diverse country, with small effects having a substantial impact on population health.

## 5. Conclusions

The analysis shows strong anti-smoking attitudes among a large, diverse sample of Aboriginal and Torres Strait Islander adults across the nation, with three-quarters of current smokers reporting a desire to quit. The analysis demonstrates that there is substantial opportunity to progress these anti-smoking attitudes into anti-smoking behaviours. While robust evidence of impact is required, these findings from the Mayi Kuwayu Study highlight the critically important need to provide accessible, comprehensive and appropriate supports for Aboriginal and Torres Strait Islander peoples to be smoke-free.

## Figures and Tables

**Table 1:** Mayi Kuwayu Study smoking-related variables used in the current analysis.

	All Participants	Current Smokers	Past Smokers
Smoking- related attitudes	Non-smokers do not miss out on gossip or yarning My community disapproves of smoking Belief that smoking is risky	Smoking has caused illness Smoking will cause illness in the future Wanting to quit smoking Reasons for wanting to stop smoking	Past smoking has caused illness Past smoking will cause illness in the future Length of time since stopped smoking Factors reported that led to quitting Methods that helped quitting
Smoking- related behaviours	Smoking status Smoking in household Smoking in car	Age at smoking initiation Reduction or cessation attempt in the past year Smoking frequency Number of cigarettes smoked daily Time to first cigarette Participation in a quit program, service or activity	Age at smoking initiation Smoking frequency Number of cigarettes smoked daily Time to first cigarette Participation in a quit program, service or activity

**Table 5:** Relationships between TIS and smoking-related attitudes and behaviours in the total sample (includes current, past, and never smokers).

	% (*n*)	Unadjusted PR (95% CI)	PR Adjusted for Age, Gender (95% CI)
**Attitudes**			
Non-smokers do not miss out on gossip or yarning			
Non-TIS	64.7 (2905)	1 (Ref)	1 (Ref)
TIS	65.6 (2137)	1.02 (0.99, 1.05)	1.01 (0.98, 1.05)
My community disapproves of smoking			
Non-TIS	67.9 (2957)	1 (Ref)	1 (Ref)
TIS	69.1 (2171)	1.02 (0.99, 1.05)	1.02 (0.99, 1.05)
Belief that smoking is risky			
Non-TIS	56.1 (2453)	1 (Ref)	1 (Ref)
TIS	55.9 (1760)	1.00 (0.96, 1.04)	1.00 (0.96, 1.04)
**Behaviours**			
Currently smoke			
Non-TIS	25.8 (1282)	1 (Ref)	1 (Ref)
TIS	26.1 (931)	1.01 (0.94, 1.09)	1.02 (0.95, 1.10)
Anyone smokes in the home			
Non-TIS	10.0 (498)	1 (Ref)	1 (Ref)
TIS	8.4 (300)	0.84 (0.73, 0.97)	0.85 (0.74, 0.97)
Anyone smokes outside the home			
Non-TIS	19.5 (970)	1 (Ref)	1 (Ref)
TIS	19.3 (686)	0.99 (0.91, 1.08)	0.98 (0.90, 1.07)
Anyone smokes in the car			
Non-TIS	8.7 (433)	1 (Ref)	1 (Ref)
TIS	7.9 (283)	0.91 (0.79, 1.05)	0.91 (0.79, 1.05)

Participants identifying as another gender are excluded from this table to protect confidentiality and avoid small cells.

**Table 6:** Relationships between TIS and smoking-related attitudes, among current smokers and among past smokers.

**Current Smoker**			
	**% (*n*)**	**Unadjusted PR (95% CI)**	**PR Adjusted for Age, Gender (95% CI)**
Smoking has made me sick			
Non-TIS	51.2 (462)	1 (Ref)	1 (Ref)
TIS	48.5 (332)	0.99 (0.90, 1.10)	1.00 (0.91, 1.11)
Smoking will make me sick in the future			
Non-TIS	88.9 (953)	1 (Ref)	1 (Ref)
TIS	91.5 (724)	1.03 (<1.00, 1.06)	1.03 (<1.00, 1.06)
Wants to quit smoking			
Non-TIS	87.4 (973)	1 (Ref)	1 (Ref)
TIS	88.0 (714)	1.00 (0.97, 1.04)	1.00 (0.97, 1.04)
Reasons for wanting to quit			
Advertising against smoking			
Non-TIS	3.7 (48)	1 (Ref)	1 (Ref)
TIS	2.5 (23)	0.66 (0.40, 1.08)	0.65 (0.40, 1.06)
Medical advice			
Non-TIS	17.2 (221)	1 (Ref)	1 (Ref)
TIS	17.2 (160)	1.00 (0.83, 1.20)	1.04 (0.86, 1.24)
My Health			
Non-TIS	55.2 (708)	1 (Ref)	1 (Ref)
TIS	56.0 (521)	1.01 (0.94, 1.09)	1.01 (0.94, 1.09)
Health of my family			
Non-TIS	23.5 (301)	1 (Ref)	1 (Ref)
TIS	24.9 (232)	1.06 (0.91, 1.23)	1.05 (0.90, 1.21)
Cost			
Non-TIS	47.9 (614)	1 (Ref)	1 (Ref)
TIS	48.3 (450)	1.00 (0.92, 1.10)	1.00 (0.92, 1.10)
Pregnancy			
Non-TIS	0.6 (8)	1 (Ref)	1 (Ref)
TIS	1.2 (11)	1.89 (0.76, 4.69)	1.65 (0.67, 4.06)
Too many non-smoking areas			
Non-TIS	4.5 (58)	1 (Ref)	1 (Ref)
TIS	4.3 (40)	0.95 (0.64, 1.41)	0.96 (0.65, 1.43)
Pressure from family or friends			
Non-TIS	14.9 (191)	1 (Ref)	1 (Ref)
TIS	14.2 (132)	0.95 (0.78, 1.17)	0.96 (0.78, 1.17)
**Past smoker**			
	**% (*n*)**	**Unadjusted PR (95% CI)**	**PR Adjusted for Age, Gender (95% CI)**
Past smoking has made me sick			
Non-TIS	34.9 (394)	1 (Ref)	1 (Ref)
TIS	35.3 (295)	1.01 (0.90, 1.14)	1.00 (0.89, 1.14)
Past smoking will make me sick in the future			
Non-TIS	54.2 (532)	1 (Ref)	1 (Ref)
TIS	56.4 (417)	1.04 (0.96, 1.13)	1.04 (0.95, 1.13)
What led you to quitting			
Advertising against smoking			
Non-TIS	5.6 (91)	1 (Ref)	1 (Ref)
TIS	7.0 (84)	1.25 (0.94, 1.66)	1.25 (0.94, 1.67)
Medical advice			
Non-TIS	15.4 (251)	1 (Ref)	1 (Ref)
TIS	15.6 (188)	1.01 (0.85, 1.20)	1.00 (0.84, 1.19)
My Health			
Non-TIS	46.2 (754)	1 (Ref)	1 (Ref)
TIS	46.8 (565)	1.01 (0.94, 1.10)	1.01 (0.94, 1.10)
Health of my family			
Non-TIS	18.2 (297)	1 (Ref)	1 (Ref)
TIS	17.6 (213)	0.97 (0.83, 1.14)	0.97 (0.83, 1.14)
Cost			
Non-TIS	28.3 (462)	1 (Ref)	1 (Ref)
TIS	28.5 (344)	1.00 (0.89, 1.13)	1.00 (0.90, 1.13)
Pregnancy			
Non-TIS	9.4 (154)	1 (Ref)	1 (Ref)
TIS	7.0 (85)	0.75 (0.58, 0.96)	0.74 (0.58, 0.94)
Too many non-smoking areas			
Non-TIS	1.6 (26)	1 (Ref)	1 (Ref)
TIS	2.1 (25)	1.30 (0.75, 2.24)	1.30 (0.76, 2.25)
Pressure from family or friends			
Non-TIS	11.2 (183)	1 (Ref)	1 (Ref)
TIS	12.8 (155)	1.15 (0.94, 1.40)	1.15 (0.94, 1.41)
What helped you quit? *			
Smoking program			
Non-TIS	3.2 (53)	1 (Ref)	1 (Ref)
TIS	2.5 (30)	0.77 (0.49, 1.19)	0.77 (0.49, 1.20)
Quitline			
Non-TIS	2.0 (32)	1 (Ref)	1 (Ref)
TIS	1.0 (12)	0.51 (0.26, 0.98)	0.52 (0.27, >1.00)
Online support			
Non-TIS	0.4 (6)	1 (Ref)	1 (Ref)
TIS	--	0.45 (0.91, 2.23)	0.46 (0.09, 2.28)
Health professional			
Non-TIS	4.7 (76)	1 (Ref)	1 (Ref)
TIS	3.4 (41)	0.73 (0.50, 1.06)	0.73 (0.50, 1.06)
Family or friends			
Non-TIS	8.8 (144)	1 (Ref)	1 (Ref)
TIS	9.4 (113)	1.06 (0.84, 1.34)	1.06 (0.84, 1.34)
Patches, gum, inhaler (NRT)			
Non-TIS	9.2 (150)	1 (Ref)	1 (Ref)
TIS	9.5 (115)	1.04 (0.82, 1.30)	1.04 (0.83, 1.31)
Stop smoking medication			
Non-TIS	8.1 (133)	1 (Ref)	1 (Ref)
TIS	6.3 (76)	0.77 (0.59, 1.01)	0.79 (0.60, 1.03)
Quit on my own			
Non-TIS	65.2 (1064)	1 (Ref)	1 (Ref)
TIS	66.6 (804)	1.02 (0.97, 1.08)	1.02 (0.96, 1.07)

Participants identifying as another gender are excluded from this table to protect confidentiality and avoid small cells. -- indicates a suppressed cell due to small numbers. * Indicates a question where multiple responses are possible. This means that the column percentages may sum to more than 100%.

**Table 7:** Relationships between TIS and smoking-related behaviours, among current smokers and among past smokers.

**Current Smokers**			
	**% (*n*)**	**Unadjusted PR (95% CI)**	**PR Adjusted for Age, Gender (95% CI)**
Age commenced smoking ≤15 years			
Non-TIS	55.5 (689)	1 (Ref)	1 (Ref)
TIS	55.9 (504)	1.00 (0.93, 1.09)	1.00 (0.93, 1.09)
Quit attempt in last 12 months			
Non-TIS	44.5 (570)	1 (Ref)	1 (Ref)
TIS	45.5 (424)	1.02 (0.93, 1.12)	1.03 (0.93, 1.13)
Reduction attempt in last 12 months			
Non-TIS	48.0 (615)	1 (Ref)	1 (Ref)
TIS	49.0 (456)	1.02 (0.94, 1.11)	1.02 (0.93, 1.11)
Smokes daily			
Non-TIS	82.2 (1029)	1 (Ref)	1 (Ref)
TIS	84.4 (774)	1.03 (0.99, 1.07)	1.03 (<1.00, 1.07)
Smoking ≥21 cigarettes per day			
Non-TIS	13.2 (160)	1 (Ref)	1 (Ref)
TIS	9.9 (87)	0.75 (0.58, 0.96)	0.79 (0.62, <1.00)
Anyone smokes in the home			
Non-TIS	27.6 (354)	1 (Ref)	1 (Ref)
TIS	21.9 (204)	0.79 (0.68, 0.92)	0.82 (0.70, 0.95)
Time to first cigarette ≤ 5 min			
Non-TIS	29.0 (359)	1 (Ref)	1 (Ref)
TIS	25.1 (228)	0.87 (0.75, <1.00)	0.87 (0.76, <1.00)
Participation in any quit smoking program or activity			
Non-TIS	10.8 (139)	1 (Ref)	1 (Ref)
TIS	12.4 (115)	1.14 (0.90, 1.44)	1.16 (0.92, 1.46)
**Past smokers**			
	**% (*n*)**	**Unadjusted PR (95% CI)**	**PR Adjusted for Age, Gender (95% CI)**
Age commenced smoking ≤15 years			
Non-TIS	52.9 (793)	1 (Ref)	1 (Ref)
TIS	50.5 (556)	0.95 (0.88, 1.03)	0.95 (0.88, 1.03)
Smoked daily			
Non-TIS	88.4 (1351)	1 (Ref)	1 (Ref)
TIS	87.5 (977)	0.99 (0.96, 1.02)	0.99 (0.96, 1.02)
Past smoking consumption ≥ 21 cigarettes			
Non-TIS	27.5 (410)	1 (Ref)	1 (Ref)
TIS	25.8 (283)	0.94 (0.82, 1.07)	0.93 (0.82, 1.05)
Length of time since quitting <5 years			
Non-TIS	26.9 (408)	1 (Ref)	1 (Ref)
TIS	28.5 (316)	1.06 (0.93, 1.20)	1.05 (0.95, 1.16)
Anyone smokes in the home			
Non-TIS	3.7 (60)	1 (Ref)	1 (Ref)
TIS	3.9 (47)	1.06 (0.73, 1.54)	1.06 (0.73, 1.54)
Past time to first cigarette ≤ 5 min			
Non-TIS	26.0 (388)	1 (Ref)	1 (Ref)
TIS	25.2 (274)	0.97 (0.85, 1.11)	0.97 (0.85, 1.11)
Participation in any quit smoking program or activity			
Non-TIS	5.8 (95)	1 (Ref)	1 (Ref)
TIS	5.9 (71)	1.01 (0.75, 1.36)	1.01 (0.75, 1.34)

Participants identifying as another gender are excluded from this table to protect confidentiality and avoid small cells.

## Data Availability

The dataset analysed during the current study is available on application to the Mayi Kuwayu Study Data Governance Committee. This governance body oversees and approves applications for data use, in order to maintain the confidentiality of participants, and ensure that all studies using the Mayi Kuwayu data are protective of Aboriginal and Torres Strait Islander data and cultures. The data application process is detailed here: mkstudy.com.au/over-view/ (last accessed 25 September 2021).

## Supplementary Materials

The following are available online at https://www.mdpi.com/article/10.3390/ijerph182010962/s1, Table S1. Smoking-related outcomes in the total sample, overall and by gender and remoteness, Table S2. Smoking-related outcomes in the total sample, overall and by age group, Table S3. Smoking-related outcomes among current smokers, overall and by gender and remoteness, Table S4. Smoking-related outcomes among current smokers, overall and by age group, Table S5. Smoking-related outcomes among past smokers, overall and by gender and remoteness, Table S6. Smoking-related outcomes among past smokers, overall and by age group, Table S7: Recoding of smoking-related attitudinal outcomes, Table S8: Recoding of smoking-related behavioural outcomes, Table S9: Associations between TIS exposure and smoking-related attitudes and behaviours in the total sample (includes current, past, and never smokers), adjusted for age, gender and remoteness, Table S10. Associations between TIS exposure and smoking-related attitudes, among current and past smokers, adjusted for age, gender and remoteness, Table S11: Associations between TIS exposure and smoking-related behaviours, among current and past smokers, adjusted for age, gender and remoteness.
