# Supplementary material for: Tobacco Related Attitudes and Behaviours in Relation to Exposure to the Tackling Indigenous Smoking Program: Evidence from the Mayi Kuwayu Study
Source: Int J Environ Res Public Health. 2021 Oct 19;18(20):10962. doi: 10.3390/ijerph182010962 (PMC8535551; doi:10.3390/ijerph182010962)
Supplement: Supplementary file 1 [file ijerph-18-10962-s001.zip › ijerph-1293154-supplementary.pdf]

Supplementary tables

Table S1. Smoking-related outcomes in the total sample^, overall and by gender and remoteness

		By gender		By remoteness					
	**Total** *N*=8540 % (*n*)	**Men** *N*=3285 % (*n*)	**Women** *N*=5255 % (*n*)	**Major City** *N*=3595 % (*n*)	**Inner regional** *N*=2449 % (*n*)	**Outer regional** *N*=1579 % (*n*)	**Remote** *N*=270 % (*n*)	**Very remote** *N*=546 % (*n*)	**Missing** *N*=101 % (*n*)
**Smoking attitudes**									
Do you agree that non-smokers miss out on gossip or yarning?									
Not at all	59.0 (5042)	59.4 (1952)	58.8 (3090)	59.6 (2144)	61.2 (1498)	60.5 (955)	52.6 (142)	46.0 (251)	51.5 (52)
A little bit	16.7 (1425)	16.1 (530)	17.0 (895)	18.2 (656)	15.9 (390)	15.7 (248)	15.2 (41)	13.7 (75)	14.9 (15)
A fair bit / A lot	15.0 (1282)	14.8 (486)	15.1 (796)	13.9 (500)	13.6 (332)	14.3 (226)	22.6 (61)	25.5 (139)	23.8 (24)
Missing	9.3 (791)	9.6 (317)	9.0 (474)	8.2 (295)	9.4 (229)	9.5 (150)	9.6 (26)	14.8 (81)	9.9 (10)
Do you agree that your community disapproves of smoking?									
Not at all	27.8 (2371)	25.9 (852)	28.9 (1519)	26.7 (959)	26.6 (652)	29.7 (469)	35.6 (96)	30.0 (164)	30.7 (31)
A little bit	23.9 (2043)	23.0 (756)	24.5 (1287)	23.1 (829)	24.6 (602)	24.3 (383)	27.4 (74)	24.2 (132)	22.8 (23)
A fair bit / A lot	36.1 (3085)	38.3 (1259)	34.7 (1826)	39.9 (1435)	36.5 (894)	32.4 (511)	24.4 (66)	26.7 (146)	32.7 (33)
Missing	12.2 (1041)	12.7 (418)	11.9 (623)	10.3 (372)	12.3 (301)	13.7 (216)	12.6 (34)	19.0 (104)	13.9 (14)
Do you agree that smoking is not that risky?									
Not at all	49.3 (4213)	46.2 (1518)	51.3 (2695)	55.7 (2001)	49.0 (1199)	44.3 (699)	42.6 (115)	28.4 (155)	34.7 (35)
A little bit	7.0 (594)	7.2 (236)	6.8 (358)	6.0 (217)	7.1 (174)	7.2 (114)	7.8 (21)	10.4 (57)	36.6 (37)
A fair bit / A lot	31.7 (2708)	34.2 (1124)	30.1 (1584)	27.8 (998)	32.1 (785)	35.1 (554)	38.1 (103)	43.0 (235)	28.7 (29)
Missing	12.0 (1025)	12.4 (407)	11.8 (618)	10.5 (379)	11.9 (291)	13.4 (212)	11.5 (31)	18.1 (99)	34.7 (35)
**Smoking behaviours**									
Smoking status									
Never smoker	40.8 (3488)	36.1 (1187)	43.8 (2301)	43.5 (1564)	39.9 (977)	37.6 (593)	38.1 (103)	39.6 (216)	34.7 (35)
Current smoker	25.9 (2213)	26.6 (874)	25.5 (1339)	21.8 (784)	25.0 (613)	28.3 (447)	34.8 (94)	43.6 (238)	36.6 (37)
Past smoker	33.2 (2839)	37.3 (1224)	30.7 (1615)	34.7 (1247)	35.1 (859)	34.1 (539)	27.0 (73)	16.8 (92)	28.7 (29)
Does anyone smoke in your home or in your car?*									
No	68.4 (5842)	69.3 (2277)	67.8 (3565)	72.7 (2613)	70.2 (1719)	67.9 (1072)	57.0 (154)	40.8 (223)	60.4 (61)
Selected 'inside the home'	9.3 (798)	10.9 (359)	8.4 (439)	8.2 (294)	9.1 (222)	8.5 (135)	13.3 (36)	17.0 (93)	17.8 (18)
Selected 'outside the home'	19.4 (1656)	16.8 (553)	21.0 (1103)	17.1 (616)	17.7 (433)	19.9 (314)	28.1 (76)	35.5 (194)	22.8 (23)
Selected 'in the car'	8.4 (716)	8.1 (266)	8.6 (450)	7.6 (274)	8.8 (216)	8.5 (134)	9.6 (26)	10.3 (56)	9.9 (10)

^ Excludes participants with other gender (*N*=9) to protect confidentiality.

* Indicates a question where multiple responses are possible. This means that the column percentages may sum to more than 100%.

Table S2. Smoking-related outcomes in the total sample^, overall and by age group

	**Total** *N*=8540 % (*n*)	**By age category** 16-24 *N*=898 % (*n*)	25-34 *N*=1134 % (*n*)	35-44 *N*=1267 % (*n*)	45-54 *N*=1637 % (*n*)	≥55 *N*=3604 % (*n*)
**Smoking attitudes**						
Do you agree that non-smokers miss out on gossip or yarning?						
Not at all	59.0 (5042)	61.0 (548)	57.4 (651)	54.8 (694)	57.0 (933)	61.5 (2216)
A little bit	16.7 (1425)	17.9 (161)	20.5 (233)	19.4 (246)	19.2 (315)	13.0 (470)
A fair bit / A lot	15.0 (1282)	13.9 (125)	15.1 (171)	18.7 (237)	15.2 (249)	13.9 (500)
Missing	9.3 (791)	7.1 (64)	7.0 (79)	7.1 (90)	8.6 (140)	11.6 (418)
Do you agree that your community disapproves of smoking?						
Not at all	27.8 (2371)	36.6 (329)	34.0 (385)	29.0 (368)	28.8 (471)	22.7 (818)
A little bit	23.9 (2043)	29.0 (260)	27.8 (315)	29.3 (371)	25.6 (419)	18.8 (678)
A fair bit / A lot	36.1 (3085)	25.7 (231)	29.6 (336)	32.8 (415)	34.6 (567)	42.6 (1536)
Missing	12.2 (1041)	8.7 (78)	8.6 (98)	8.9 (113)	11.0 (180)	15.9 (572)
Do you agree that smoking is not that risky?						
Not at all	49.3 (4213)	54.5 (489)	58.3 (661)	56.3 (713)	50.0 (819)	42.5 (1531)
A little bit	7.0 (594)	12.1 (109)	7.9 (90)	6.9 (87)	7.1 (117)	5.3 (191)
A fair bit / A lot	31.7 (2708)	25.2 (226)	25.1 (285)	27.6 (350)	32.1 (526)	36.7 (1321)
Missing	12.0 (1025)	8.2 (74)	8.6 (98)	9.2 (117)	10.7 (175)	15.6 (561)
**Smoking behaviours**						
Smoking status						
Never smoker	40.8 (3488)	59.6 (535)	47.7 (541)	38.2 (484)	36.0 (590)	37.1 (1338)
Current smoker	25.9 (2213)	27.7 (249)	31.3 (355)	32.8 (415)	32.4 (530)	18.4 (664)
Past smoker	33.2 (2839)	12.7 (114)	21.0 (238)	29.0 (368)	31.6 (517)	44.5 (1602)
Does anyone smoke in your home or in your car?*						
No	68.4 (5842)	52.1 (468)	62.9 (713)	68.9 (873)	65.4 (1070)	75.4 (2718)
Selected 'inside the home'	9.3 (798)	11.7 (105)	7.9 (90)	9.1 (115)	11.9 (195)	8.1 (293)
Selected 'outside the home'	19.4 (1656)	33.1 (297)	25.4 (288)	19.1 (242)	20.2 (330)	13.8 (499)
Selected 'in the car'	8.4 (716)	12.9 (116)	10.0 (113)	7.7 (98)	9.9 (162)	6.3 (227)

^ Excludes participants with other gender (*N*=9) to protect confidentiality.

* Indicates a question where multiple responses are possible. This means that the column percentages may sum to more than 100%.

Table S3. Smoking-related outcomes among current smokers^, overall and by gender and remoteness

	**Total Current Smoker** *N*=2213 % (*n*)	**By gender**		**By remoteness**					
		**Men** *N*=874 % (*n*)	**Women** *N*=1339 % (*n*)	**Major City** *N*=784 % (*n*)	**Inner regional** *N*=613 % (*n*)	**Outer regional** *N*=447 % (*n*)	**Remote** *N*=94 % (*n*)	**Very remote** *N*=238 % (*n*)	**Missing** *N*=37 % (*n*)
**Smoking attitudes**									
Do you think your smoking has made you sick?									
No	36.8 (814)	36.8 (322)	36.7 (492)	36.9 (289)	41.1 (252)	36.9 (165)	33.0 (31)	25.6 (61)	43.2 (16)
Yes	34.9 (773)	35.8 (313)	34.4 (460)	35.7 (280)	33.1 (203)	34.0 (152)	38.3 (36)	39.1 (93)	24.3 (9)
Unsure	25.9 (574)	24.8 (217)	26.7 (357)	25.6 (201)	24.1 (148)	27.3 (122)	26.6 (25)	27.7 (66)	32.4 (12)
Missing	2.3 (52)	2.5 (22)	2.2 (30)	1.8 (14)	1.6 (10)	1.8 (8)	2.1 (2)	7.6 (18)	43.2 (16)
Do you think your smoking will make you sick in the future?									
Not at all	8.4 (186)	8.9 (78)	8.1 (108)	8.7 (68)	8.0 (49)	8.5 (38)	8.5 (8)	6.3 (15)	21.6 (8)
A little bit	20.2 (446)	19.6 (171)	20.5 (275)	21.6 (169)	21.0 (129)	21.3 (95)	17.0 (16)	14.3 (34)	8.1 (3)
A fair bit / A lot	55.6 (1231)	55.7 (487)	55.6 (744)	55.5 (435)	56.1 (344)	54.4 (243)	58.5 (55)	56.7 (135)	51.4 (19)
Unsure	14.0 (310)	13.7 (120)	14.2 (190)	13.3 (104)	13.7 (84)	14.1 (63)	12.8 (12)	16.8 (40)	18.9 (7)
Missing	1.8 (40)	2.1 (18)	1.6 (22)	1.0 (8)	1.1 (7)	1.8 (8)	3.2 (3)	5.9 (14)	21.6 (8)
Do you want to quit smoking?									
Not at all	10.7 (237)	12.0 (105)	9.9 (132)	11.6 (91)	11.3 (69)	9.8 (44)	14.9 (14)	6.7 (16)	8.1 (3)
A little bit	21.7 (480)	17.8 (156)	24.2 (324)	21.8 (171)	24.8 (152)	20.4 (91)	14.9 (14)	18.5 (44)	21.6 (8)
A fair bit / A lot	54.5 (1207)	55.6 (486)	53.8 (721)	57.4 (450)	54.0 (331)	55.7 (249)	52.1 (49)	46.2 (110)	48.6 (18)
Unsure	10.8 (240)	11.6 (101)	10.4 (139)	7.7 (60)	8.5 (52)	12.5 (56)	16.0 (15)	21.0 (50)	18.9 (7)
Missing	2.2 (49)	3.0 (26)	1.7 (23)	1.5 (12)	1.5 (9)	1.6 (7)	2.1 (2)	7.6 (18)	2.7 (1)
Why do you want to quit?*									
Advertising against smoking	3.2 (71)	2.3 (20)	3.8 (51)	2.9 (23)	3.4 (21)	2.9 (13)	--	3.8 (9)	8.1 (3)
Medical advice	17.2 (381)	18.1 (158)	16.7 (223)	18.5 (145)	20.1 (123)	15.0 (67)	14.9 (14)	11.8 (28)	10.8 (4)
My Health	55.5 (1229)	54.2 (474)	56.4 (755)	59.1 (463)	53.7 (329)	56.4 (252)	51.1 (48)	49.6 (118)	51.4 (19)
Health of my family	24.1 (533)	20.3 (177)	26.6 (356)	25.3 (198)	24.8 (152)	21.9 (98)	19.1 (18)	26.1 (62)	13.5 (5)
Cost	48.1 (1064)	46.7 (408)	49.0 (656)	52.0 (408)	52.2 (320)	47.2 (211)	35.1 (33)	30.3 (72)	54.1 (20)
Pregnancy	0.9 (19)	--	1.3 (18)	1.0 (8)	1.3 (8)	--	--	--	0 (0)
Too many non-smoking areas	4.4 (98)	3.8 (33)	4.9 (65)	4.8 (38)	4.9 (30)	4.3 (19)	--	--	5.4 (2)
Pressure from family or friends	14.6 (323)	14.3 (125)	14.8 (198)	15.6 (122)	14.2 (87)	12.3 (55)	18.1 (17)	15.1 (36)	16.2 (6)
Other	6.2 (138)	5.9 (52)	6.4 (86)	7.0 (55)	5.9 (36)	4.5 (20)	--	8.4 (20)	8.1 (3)
Missing or indicated does not want to quit	15.5 (344)	17.3 (151)	14.4 (193)	14.5 (114)	14.0 (86)	--	25.5 (24)	21.4 (51)	13.5 (5)
**Smoking behaviours**									
How old were you when you started smoking?									

1-15 years old	53.9 (1193)	56.2 (491)	52.4 (702)	58.7 (460)	61.7 (378)	50.3 (225)	39.4 (37)	29.4 (70)	62.2 (23)
16-18 years old	28.8 (637)	24.6 (215)	31.5 (422)	26.1 (205)	24.6 (151)	32.0 (143)	45.7 (43)	36.1 (86)	24.3 (9)
19-24 years old	8.9 (196)	9.2 (80)	8.7 (116)	8.2 (64)	6.4 (39)	7.8 (35)	10.6 (10)	19.7 (47)	2.7 (1)
25-34 years old	3.4 (75)	4.5 (39)	2.7 (36)	2.3 (18)	4.2 (26)	4.3 (19)	--	4.2 (10)	2.7 (1)
≥35 years old	1.9 (42)	1.8 (16)	1.9 (26)	2.6 (20)	1.3 (8)	2.5 (11)	--	--	2.7 (1)
Missing	3.2 (70)	3.8 (33)	2.8 (37)	2.2 (17)	1.8 (11)	3.1 (14)	3.2 (3)	--	5.4 (2)
In the past year, have you tried to quit or reduce the amount you smoke?*									
Tried to quit smoking	44.9 (994)	42.2 (369)	46.7 (625)	44.8 (351)	46.5 (285)	41.6 (186)	41.5 (39)	50.0 (119)	37.8 (14)
Tried to reduce smoking	48.4 (1071)	44.4 (388)	51.0 (683)	53.3 (418)	50.9 (312)	49.4 (221)	34.0 (32)	31.5 (75)	35.1 (13)
Have not tried to quit or reduce smoking	18.0 (399)	21.3 (186)	15.9 (213)	17.0 (133)	17.5 (107)	17.7 (79)	26.6 (25)	18.1 (43)	32.4 (12)
Missing	2.8 (62)	3.2 (28)	2.5 (34)	1.9 (15)	1.8 (11)	2.5 (11)	4.3 (4)	7.6 (18)	32.4 (12)
How often do you smoke?									
Less than weekly	5.2 (115)	4.8 (42)	5.5 (73)	5.2 (41)	3.4 (21)	4.0 (18)	--	11.3 (27)	8.1 (3)
Weekly (not every day)	11.4 (252)	12.0 (105)	11.0 (147)	9.7 (76)	7.5 (46)	11.2 (50)	21.3 (20)	23.9 (57)	8.1 (3)
Every day	81.5 (1803)	80.8 (706)	81.9 (1097)	83.9 (658)	87.6 (537)	83.4 (373)	70.2 (66)	58.4 (139)	81.1 (30)
Missing	1.9 (43)	2.4 (21)	1.6 (22)	1.1 (9)	1.5 (9)	1.3 (6)	--	6.3 (15)	2.7 (1)
How many cigarettes do you usually smoke in one day?									
1-10 cigarettes per day	53.1 (1176)	47.5 (415)	56.8 (761)	51.7 (405)	50.1 (307)	51.7 (231)	69.1 (65)	63.9 (152)	43.2 (16)
11-20 cigarettes per day	30.3 (670)	32.8 (287)	28.6 (383)	32.0 (251)	35.6 (218)	32.2 (144)	14.9 (14)	13.4 (32)	29.7 (11)
21-30 cigarettes per day	8.9 (198)	11.0 (96)	7.6 (102)	10.2 (80)	7.7 (47)	8.3 (37)	9.6 (9)	8.0 (19)	16.2 (6)
≥31 cigarettes per day	2.2 (49)	3.0 (26)	1.7 (23)	2.7 (21)	2.9 (18)	2.0 (9)	--	--	0 (0)
Missing or less than daily	5.4 (120)	5.7 (50)	5.2 (70)	3.4 (27)	3.8 (23)	5.8 (26)	--	--	10.8 (4)
How soon after waking do you usually have your first cigarette?									
5 minutes or less	26.5 (587)	29.6 (259)	24.5 (328)	27.4 (215)	25.4 (156)	26.4 (118)	23.4 (22)	26.9 (64)	32.4 (12)
6-30 minutes	36.6 (809)	34.2 (299)	38.1 (510)	35.3 (277)	40.5 (248)	36.2 (162)	38.3 (36)	29.4 (70)	43.2 (16)
31-60 minutes	16.1 (356)	16.7 (146)	15.7 (210)	17.2 (135)	15.7 (96)	17.2 (77)	14.9 (14)	12.2 (29)	13.5 (5)
61 or more minutes	9.3 (205)	8.2 (72)	9.9 (133)	7.7 (60)	9.5 (58)	10.3 (46)	16.0 (15)	10.9 (26)	0 (0)
Don't smoke every day	8.5 (187)	7.7 (67)	9.0 (120)	10.1 (79)	7.8 (48)	7.4 (33)	--	8.8 (21)	8.1 (3)
Missing	3.1 (69)	3.5 (31)	2.8 (38)	2.3 (18)	1.1 (7)	2.5 (11)	--	11.8 (28)	2.7 (1)
Have you ever participated in any Quit Smoking Program, Service or Activity?									
Not reported	88.5 (1959)	89.5 (782)	87.9 (1177)	86.7 (680)	85.5 (524)	91.3 (408)	91.5 (86)	95.0 (226)	94.6 (35)
Reported	11.5 (254)	10.5 (92)	12.1 (162)	13.3 (104)	14.5 (89)	8.7 (39)	8.5 (8)	5.0 (12)	5.4 (2)

^ Excludes participants with other gender (*N*=9) to protect confidentiality.

* Indicates a question where multiple responses are possible. This means that the column percentages may sum to more than 100%.

-- indicates a cell that was suppressed due to small numbers.

Table S4. Smoking-related outcomes among current smokers^, overall and by age group

	Total Current smokers *N*=2213 % (*n*)	By age group (years) 16-24 *N*=249 % (*n*)	25-34 *N*=355 % (*n*)	35-44 *N*=415 % (*n*)	45-54 *N*=530 % (*n*)	≥55 *N*=664 % (*n*)
**Smoking attitudes**						
Do you think your smoking has made you sick?						
No	36.8 (814)	42.2 (105)	36.6 (130)	35.7 (148)	35.3 (187)	36.7 (244)
Yes	34.9 (773)	29.7 (74)	32.1 (114)	34.5 (143)	39.1 (207)	35.4 (235)
Unsure	25.9 (574)	26.1 (65)	28.7 (102)	27.2 (113)	24.0 (127)	25.2 (167)
Missing	2.3 (52)	2.0 (5)	2.5 (9)	2.7 (11)	1.7 (9)	2.7 (18)
Do you think your smoking will make you sick in the future?						
Not at all	8.4 (186)	8.0 (20)	7.3 (26)	6.0 (25)	9.4 (50)	9.8 (65)
A little bit	20.2 (446)	24.5 (61)	21.1 (75)	21.7 (90)	17.5 (93)	19.1 (127)
A fair bit / A lot	55.6 (1231)	53.8 (134)	56.6 (201)	59.0 (245)	57.9 (307)	51.8 (344)
Unsure	14.0 (310)	12.4 (31)	13.5 (48)	11.1 (46)	13.2 (70)	17.3 (115)
Missing	1.8 (40)	1.2 (3)	1.4 (5)	2.2 (9)	1.9 (10)	2.0 (13)
Do you want to quit smoking?						
Not at all	10.7 (237)	8.4 (21)	8.2 (29)	7.0 (29)	12.3 (65)	14.0 (93)
A little bit	21.7 (480)	29.3 (73)	23.9 (85)	21.2 (88)	17.9 (95)	20.9 (139)
A fair bit / A lot	54.5 (1207)	43.8 (109)	54.9 (195)	58.3 (242)	58.7 (311)	52.7 (350)
Unsure	10.8 (240)	16.9 (42)	10.4 (37)	10.8 (45)	9.4 (50)	9.9 (66)
Missing	2.2 (49)	1.6 (4)	2.5 (9)	2.7 (11)	1.7 (9)	2.4 (16)
Why do you want to quit?*						
Advertising against smoking	3.2 (71)	2.4 (6)	3.4 (12)	3.1 (13)	4.3 (23)	2.6 (17)
Medical advice	17.2 (381)	8.8 (22)	10.4 (37)	11.6 (48)	20.9 (111)	24.5 (163)
My Health	55.5 (1229)	55.0 (137)	57.7 (205)	55.4 (230)	56.2 (298)	54.1 (359)
Health of my family	24.1 (533)	21.7 (54)	33.8 (120)	35.2 (146)	20.6 (109)	15.7 (104)
Cost	48.1 (1064)	42.6 (106)	50.7 (180)	50.1 (208)	47.5 (252)	47.9 (318)
Pregnancy	0.9 (19)	3.2 (8)	1.7 (6)	--	--	--
Too many non-smoking areas	4.4 (98)	--	3.1 (11)	5.8 (24)	4.0 (21)	5.7 (38)
Pressure from family or friends	14.6 (323)	12.9 (32)	12.7 (45)	14.9 (62)	15.3 (81)	15.5 (103)
Other	6.2 (138)	7.2 (18)	5.9 (21)	7.2 (30)	6.4 (34)	5.3 (35)
Missing or indicated does not want to quit	15.5 (344)	--	13.2 (47)	--	--	--
**Smoking behaviours**						
How old were you when you started smoking?						
1-15 years old	53.9 (1193)	53.0 (132)	46.5 (165)	51.1 (212)	57.7 (306)	56.9 (378)
16-18 years old	28.8 (637)	38.6 (96)	34.9 (124)	31.8 (132)	24.5 (130)	23.3 (155)
19-24 years old	8.9 (196)	5.2 (13)	12.7 (45)	9.6 (40)	9.4 (50)	7.2 (48)

25-34 years old	3.4 (75)	--	2.0 (7)	2.7 (11)	3.6 (19)	5.7 (38)
≥35 years old	1.9 (42)	--	--	1.4 (6)	3.0 (16)	3.0 (20)
Missing	3.2 (70)	3.2 (8)	--	3.4 (14)	1.7 (9)	3.8 (25)
In the past year, have you tried to quit or reduce the amount you smoke?*						
Tried to quit smoking	44.9 (994)	43.8 (109)	45.9 (163)	45.5 (189)	42.3 (224)	46.5 (309)
Tried to reduce smoking	48.4 (1071)	51.4 (128)	50.7 (180)	47.0 (195)	48.1 (255)	47.1 (313)
Have not tried to quit or reduce smoking	18.0 (399)	16.9 (42)	16.6 (59)	17.3 (72)	20.9 (111)	17.3 (115)
Missing	2.8 (62)	1.6 (4)	2.8 (10)	4.3 (18)	1.5 (8)	3.3 (22)
How often do you smoke?						
Less than weekly	5.2 (115)	10.8 (27)	7.6 (27)	4.1 (17)	4.7 (25)	2.9 (19)
Weekly (not every day)	11.4 (252)	17.3 (43)	15.2 (54)	12.8 (53)	9.8 (52)	7.5 (50)
Every day	81.5 (1803)	69.1 (172)	74.4 (264)	81.9 (340)	84.3 (447)	87.3 (580)
Missing	1.9 (43)	2.8 (7)	2.8 (10)	1.2 (5)	1.1 (6)	2.3 (15)
How many cigarettes do you usually smoke in one day?						
1-10 cigarettes per day	53.1 (1176)	76.7 (191)	65.6 (233)	54.5 (226)	45.1 (239)	43.2 (287)
11-20 cigarettes per day	30.3 (670)	10.8 (27)	22.8 (81)	32.3 (134)	36.2 (192)	35.5 (236)
21-30 cigarettes per day	8.9 (198)	4.4 (11)	3.4 (12)	6.3 (26)	11.3 (60)	13.4 (89)
≥31 cigarettes per day	2.2 (49)	--	--	2.2 (9)	2.6 (14)	3.0 (20)
Missing or less than daily	5.4 (120)	--	--	4.8 (20)	4.7 (25)	4.8 (32)
How soon after waking do you usually have your first cigarette?						
5 minutes or less	26.5 (587)	17.7 (44)	23.9 (85)	28.4 (118)	29.1 (154)	26.5 (176)
6-30 minutes	36.6 (809)	21.7 (54)	33.8 (120)	34.2 (142)	39.4 (209)	38.6 (256)
31-60 minutes	16.1 (356)	32.9 (82)	16.6 (59)	17.3 (72)	14.0 (74)	16.9 (112)
61 or more minutes	9.3 (205)	15.7 (39)	12.1 (43)	8.4 (35)	8.3 (44)	8.9 (59)
Don't smoke every day	8.5 (187)	9.6 (24)	8.2 (29)	8.4 (35)	7.2 (38)	6.2 (41)
Missing	3.1 (69)	2.4 (6)	5.4 (19)	3.1 (13)	2.1 (11)	3.0 (20)
Have you ever participated in any Quit Smoking Program, Service or Activity?						
No	88.5 (1959)	96.8 (241)	92.7 (329)	87.5 (363)	86.0 (456)	85.8 (570)
Yes	11.5 (254)	3.2 (8)	7.3 (26)	12.5 (52)	14.0 (74)	14.2 (94)

^ Excludes participants with other gender (*N*=9) to protect confidentiality.

* Indicates a question where multiple responses are possible. This means that the column percentages may sum to more than 100%.

-- indicates a cell that was suppressed due to small numbers.

Table S5. Smoking-related outcomes among past smokers^, overall and by gender and remoteness

	Total Sample N=2839 % (n)	By gender		By remoteness					
		Men N=1224 % (n)	Women N=1615 % (n)	Major City N=1247 % (n)	Inner regional N=859 % (n)	Outer regional N=539 % (n)	Remote N=73 % (n)	Very remote N=92 % (n)	Missing N=29 % (n)
**Smoking attitudes**									
Do you think your past smoking has made you sick?									
No	44.9 (1276)	40.4 (494)	48.4 (782)	45.2 (564)	47.1 (405)	43.4 (234)	35.6 (26)	39.1 (36)	37.9 (11)
Yes	24.3 (689)	27.4 (335)	21.9 (354)	24.1 (301)	22.2 (191)	25.2 (136)	30.1 (22)	32.6 (30)	31.0 (9)
Unsure	23.1 (655)	23.2 (284)	23.0 (371)	23.7 (296)	23.9 (205)	21.9 (118)	24.7 (18)	13.0 (12)	20.7 (6)
Missing	7.7 (219)	9.1 (111)	6.7 (108)	6.9 (86)	6.8 (58)	9.5 (51)	9.6 (7)	15.2 (14)	10.3 (3)
Do you think your past smoking will make you sick in the future?									
Not at all	27.2 (772)	25.3 (310)	28.6 (462)	27.3 (341)	27.9 (240)	26.2 (141)	23.3 (17)	30.4 (28)	17.2 (5)
A little bit	20.7 (587)	20.3 (248)	21.0 (339)	21.1 (263)	21.4 (184)	18.9 (102)	17.8 (13)	19.6 (18)	24.1 (7)
A fair bit / A lot	12.8 (362)	14.1 (173)	11.7 (189)	12.8 (160)	12.6 (108)	11.3 (61)	20.5 (15)	15.2 (14)	13.8 (4)
Unsure	32.5 (922)	32.6 (399)	32.4 (523)	32.4 (404)	32.6 (280)	34.7 (187)	27.4 (20)	23.9 (22)	31.0 (9)
Missing	6.9 (196)	7.7 (94)	6.3 (102)	6.3 (79)	5.5 (47)	8.9 (48)	11.0 (8)	10.9 (10)	13.8 (4)
How long ago did you quit?									
1 to 5 months	5.6 (160)	4.8 (59)	6.3 (101)	5.1 (63)	6.3 (54)	5.0 (27)	--	9.8 (9)	10.3 (3)
6 months to a year	3.5 (98)	2.6 (32)	4.1 (66)	3.4 (43)	3.4 (29)	3.5 (19)	--	--	3.4 (1)
1 to 2 years	6.0 (170)	4.7 (58)	6.9 (112)	6.8 (85)	4.5 (39)	5.0 (27)	12.3 (9)	8.7 (8)	6.9 (2)
2 to 5 years	10.4 (296)	8.4 (103)	12.0 (193)	9.4 (117)	10.6 (91)	13.0 (70)	11.0 (8)	9.8 (9)	3.4 (1)
More than 5 years	66.9 (1898)	70.2 (859)	64.3 (1039)	68.5 (854)	68.8 (591)	64.0 (345)	54.8 (40)	54.3 (50)	62.1 (18)
Missing	7.6 (217)	9.2 (113)	6.4 (104)	6.8 (85)	6.4 (55)	9.5 (51)	11.0 (8)	15.2 (14)	13.8 (4)
What led you to quit?*									
Advertising against smoking	6.2 (175)	6.5 (79)	5.9 (96)	7.3 (91)	4.9 (42)	6.1 (33)	--	--	6.9 (2)
Medical advice	15.5 (439)	19.8 (242)	12.2 (197)	13.6 (169)	16.5 (142)	17.8 (96)	19.2 (14)	10.9 (10)	27.6 (8)
My Health	46.5 (1319)	45.1 (552)	47.5 (767)	46.8 (584)	47.5 (408)	46.2 (249)	42.5 (31)	39.1 (36)	37.9 (11)
Health of my family	18.0 (510)	19.1 (234)	17.1 (276)	18.9 (236)	17.6 (151)	17.4 (94)	16.4 (12)	14.1 (13)	13.8 (4)
Cost	28.4 (806)	28.5 (349)	28.3 (457)	27.7 (346)	30.8 (265)	27.5 (148)	24.7 (18)	28.3 (26)	10.3 (3)
Pregnancy	8.4 (239)	--	14.5 (234)	9.7 (121)	7.2 (62)	8.2 (44)	--	--	10.3 (3)
Too many non-smoking areas	1.8 (51)	1.6 (20)	1.9 (31)	1.4 (17)	2.3 (20)	1.9 (10)	--	--	0 (0)
Pressure from family or friends	11.9 (338)	12.2 (149)	11.7 (189)	13.5 (168)	10.8 (93)	10.8 (58)	8.2 (6)	9.8 (9)	13.8 (4)
Other	20.1 (572)	19.9 (243)	20.4 (329)	19.9 (248)	19.9 (171)	20.6 (111)	26.0 (19)	16.3 (15)	27.6 (8)
Missing	7.5 (212)	--	5.9 (96)	6.5 (81)	5.8 (50)	10.0 (54)	12.3 (9)	16.3 (15)	89.7 (26)
What helped you quit?*									
Smoking program	2.9 (83)	2.4 (29)	3.3 (54)	2.9 (36)	3.3 (28)	2.4 (13)	--	--	6.9 (2)
Quitline	1.5 (44)	1.0 (12)	2.0 (32)	1.8 (22)	1.6 (14)	1.3 (7)	--	--	27.6 (8)

Online support	0.3 (8)	0.2 (2)	--	--	0.1 (1)	--	--	--	0 (0)
Health professional	4.1 (117)	4.2 (51)	4.1 (66)	3.8 (47)	5.0 (43)	3.3 (18)	--	--	6.9 (2)
Family or friends	9.1 (257)	9.3 (114)	8.9 (143)	9.6 (120)	8.4 (72)	10.0 (54)	--	--	6.9 (2)
Patches, gum, inhaler (NRT)	9.3 (265)	9.6 (117)	9.2 (148)	10.2 (127)	10.8 (93)	6.3 (34)	--	--	13.8 (4)
Stop smoking medication	7.4 (209)	6.4 (78)	8.1 (131)	6.7 (84)	9.5 (82)	6.1 (33)	--	--	3.4 (1)
Quit on my own	65.8 (1868)	66.1 (809)	65.6 (1059)	66.3 (827)	63.2 (543)	69.4 (374)	63.0 (46)	68.5 (63)	51.7 (15)
Other	12.5 (354)	10.9 (133)	13.7 (221)	11.4 (142)	13.9 (119)	13.9 (75)	9.6 (7)	--	24.1 (7)
Missing	0 (0)	0 (0)	0 (0)	0 (0)	0 (0)	0 (0)	0 (0)	0 (0)	0 (0)
**Smoking behaviours**									
How old were you when you started smoking?									
Under 15 years old	47.5 (1349)	50.2 (615)	45.4 (734)	48.4 (604)	50.8 (436)	43.8 (236)	38.4 (28)	33.7 (31)	48.3 (14)
16-18 years old	29.8 (845)	27.5 (337)	31.5 (508)	30.3 (378)	28.9 (248)	30.4 (164)	26.0 (19)	32.6 (30)	20.7 (6)
19-24 years old	9.7 (276)	8.5 (104)	10.7 (172)	9.9 (123)	9.1 (78)	9.3 (50)	17.8 (13)	9.8 (9)	10.3 (3)
25-34 years old	3.3 (95)	2.9 (35)	3.7 (60)	3.5 (44)	2.6 (22)	3.7 (20)	--	--	6.9 (2)
≥35 years old	1.2 (34)	0.8 (10)	1.5 (24)	1.0 (12)	1.4 (12)	1.1 (6)	--	--	3.4 (1)
Missing	8.5 (240)	10.0 (123)	7.2 (117)	6.9 (86)	7.3 (63)	11.7 (63)	11.0 (8)	18.5 (17)	10.3 (3)
When you used to smoke, how often did you smoke?									
Less than weekly	6.8 (194)	4.6 (56)	8.5 (138)	6.4 (80)	5.6 (48)	8.7 (47)	9.6 (7)	12.0 (11)	3.4 (1)
Weekly (not every day)	11.2 (317)	9.2 (113)	12.6 (204)	11.0 (137)	8.7 (75)	14.1 (76)	16.4 (12)	16.3 (15)	6.9 (2)
Every day	75.2 (2134)	78.4 (960)	72.7 (1174)	76.3 (952)	80.1 (688)	68.8 (371)	64.4 (47)	58.7 (54)	75.9 (22)
Missing	6.8 (194)	7.8 (95)	6.1 (99)	6.3 (78)	5.6 (48)	8.3 (45)	9.6 (7)	13.0 (12)	13.8 (4)
When you used to smoke, how many cigarettes did you usually smoke in one day?									
1-10 cigarettes per day	39.7 (1128)	29.2 (358)	47.7 (770)	41.7 (520)	37.5 (322)	40.8 (220)	38.4 (28)	34.8 (32)	20.7 (6)
11-20 cigarettes per day	27.1 (768)	30.1 (369)	24.7 (399)	28.1 (350)	28.4 (244)	23.9 (129)	19.2 (14)	28.3 (26)	17.2 (5)
21-30 cigarettes per day	15.6 (443)	19.6 (240)	12.6 (203)	14.8 (185)	17.3 (149)	14.5 (78)	16.4 (12)	10.9 (10)	31.0 (9)
≥31 cigarettes per day	8.8 (250)	11.2 (137)	7.0 (113)	7.7 (96)	9.9 (85)	9.3 (50)	13.7 (10)	--	17.2 (5)
Missing or less than daily	8.8 (250)	9.8 (120)	8.0 (130)	7.7 (96)	6.9 (59)	11.5 (62)	12.3 (9)	--	13.8 (4)
How soon after waking did you usually have your first cigarette?									
5 minutes or less	13.8 (393)	10.9 (134)	16.0 (259)	13.2 (164)	11.1 (95)	17.6 (95)	23.3 (17)	19.6 (18)	13.8 (4)
6-30 minutes	23.3 (662)	25.7 (314)	21.5 (348)	21.9 (273)	25.1 (216)	23.9 (129)	19.2 (14)	21.7 (20)	34.5 (10)
31-60 minutes	26.7 (759)	26.8 (328)	26.7 (431)	28.7 (358)	27.1 (233)	23.0 (124)	28.8 (21)	20.7 (19)	13.8 (4)
61 or more minutes	14.1 (399)	13.2 (162)	14.7 (237)	13.7 (171)	15.3 (131)	14.3 (77)	11.0 (8)	7.6 (7)	17.2 (5)
Don't smoke every day	13.0 (370)	12.8 (157)	13.2 (213)	15.2 (189)	13.3 (114)	9.1 (49)	--	12.0 (11)	10.3 (3)
Missing	9.0 (256)	10.5 (129)	7.9 (127)	7.4 (92)	8.1 (70)	12.1 (65)	--	18.5 (17)	10.3 (3)
Have you ever participated in any Quit Smoking Program, Service or Activity?									
No	94.2 (2673)	95.1 (1164)	93.4 (1509)	95.5 (1191)	92.4 (794)	94.8 (511)	--	--	82.8 (24)
Yes	5.8 (166)	4.9 (60)	6.6 (106)	4.5 (56)	7.6 (65)	5.2 (28)	--	--	17.2 (5)

^ Excludes participants with other gender (*N*=9) to protect confidentiality.

* Indicates a question where multiple responses are possible. This means that the column percentages may sum to more than 100%.

-- indicates a cell that was suppressed due to small numbers.

Table S6. Smoking-related outcomes among past smokers^, overall and by age group

	Total Sample *N*=2839 % (*n*)	By age group (years)				
		16-24 *N*=114 % (*n*)	25-34 *N*=238 % (*n*)	35-44 *N*=368 % (*n*)	45-54 *N*=517 % (*n*)	≥55 *N*=1602 % (*n*)
**Smoking attitudes**						
Do you think your past smoking has made you sick?						
No	44.9 (1276)	59.6 (68)	48.7 (116)	43.5 (160)	46.2 (239)	43.3 (693)
Yes	24.3 (689)	11.4 (13)	23.9 (57)	24.7 (91)	21.5 (111)	26.0 (417)
Unsure	23.1 (655)	21.1 (24)	21.0 (50)	26.6 (98)	25.1 (130)	22.0 (353)
Missing	7.7 (219)	7.9 (9)	6.3 (15)	5.2 (19)	7.2 (37)	8.7 (139)
Do you think your past smoking will make you sick in the future?						
Not at all	27.2 (772)	34.2 (39)	28.2 (67)	25.3 (93)	24.8 (128)	27.8 (445)
A little bit	20.7 (587)	20.2 (23)	30.7 (73)	26.4 (97)	22.4 (116)	17.4 (278)
A fair bit / A lot	12.8 (362)	8.8 (10)	10.9 (26)	11.7 (43)	9.7 (50)	14.5 (233)
Unsure	32.5 (922)	30.7 (35)	26.1 (62)	31.8 (117)	36.4 (188)	32.5 (520)
Missing	6.9 (196)	6.1 (7)	4.2 (10)	4.9 (18)	6.8 (35)	7.9 (126)
How long ago did you quit?						
1 to 5 months	5.6 (160)	28.1 (32)	13.0 (31)	8.4 (31)	5.4 (28)	2.4 (38)
6 months to a year	3.5 (98)	16.7 (19)	9.2 (22)	4.6 (17)	2.3 (12)	1.7 (28)
1 to 2 years	6.0 (170)	22.8 (26)	17.2 (41)	6.8 (25)	4.4 (23)	3.4 (55)
2 to 5 years	10.4 (296)	18.4 (21)	20.2 (48)	15.2 (56)	10.6 (55)	7.2 (116)
More than 5 years	66.9 (1898)	7.0 (8)	33.2 (79)	59.8 (220)	70.0 (362)	76.7 (1229)
Missing	7.6 (217)	7.0 (8)	7.1 (17)	5.2 (19)	7.2 (37)	8.5 (136)
What led you to quit?*						
Advertising against smoking	6.2 (175)	--	5.0 (12)	6.0 (22)	7.0 (36)	6.2 (100)
Medical advice	15.5 (439)	7.0 (8)	9.7 (23)	9.0 (33)	13.9 (72)	18.9 (303)
My Health	46.5 (1319)	36.8 (42)	46.6 (111)	53.5 (197)	45.5 (235)	45.8 (734)
Health of my family	18.0 (510)	13.2 (15)	20.6 (49)	20.9 (77)	20.9 (108)	16.3 (261)
Cost	28.4 (806)	29.8 (34)	33.2 (79)	32.3 (119)	30.8 (159)	25.9 (415)
Pregnancy	8.4 (239)	14.0 (16)	26.1 (62)	12.0 (44)	9.9 (51)	4.1 (66)
Too many non-smoking areas	1.8 (51)	--	--	1.6 (6)	1.9 (10)	2.0 (32)
Pressure from family or friends	11.9 (338)	13.2 (15)	12.2 (29)	13.9 (51)	14.5 (75)	10.5 (168)
Other	20.1 (572)	28.1 (32)	16.4 (39)	22.6 (83)	22.6 (117)	18.8 (301)
Missing	7.5 (212)	6.1 (7)	--	4.6 (17)	7.0 (36)	8.6 (138)
What helped you quit?*						

Smoking program	2.9 (83)	--	2.9 (7)	4.3 (16)	2.7 (14)	2.8 (45)
Quitline	1.5 (44)	--	--	3.3 (12)	1.9 (10)	1.3 (21)
Online support	0.3 (8)	--	--	--	--	--
Health professional	4.1 (117)	--	2.9 (7)	4.6 (17)	3.3 (17)	4.6 (74)
Family or friends	9.1 (257)	18.4 (21)	11.8 (28)	13.9 (51)	8.5 (44)	7.1 (113)
Patches, gum, inhaler (NRT)	9.3 (265)	--	9.2 (22)	11.4 (42)	9.1 (47)	9.5 (152)
Stop smoking medication	7.4 (209)	--	4.6 (11)	9.8 (36)	10.3 (53)	6.6 (105)
Quit on my own	65.8 (1868)	76.3 (87)	68.9 (164)	63.6 (234)	65.4 (338)	65.2 (1045)
Other	12.5 (354)	7.9 (9)	13.4 (32)	16.6 (61)	13.3 (69)	11.4 (183)
Missing	0 (0)	0 (0)	0 (0)	0 (0)	0 (0)	0 (0)
**Smoking behaviours**						
How old were you when you started smoking?						
Under 15 years old	47.5 (1349)	43.9 (50)	44.5 (106)	46.5 (171)	49.9 (258)	47.7 (764)
16-18 years old	29.8 (845)	37.7 (43)	34.0 (81)	35.9 (132)	26.9 (139)	28.1 (450)
19-24 years old	9.7 (276)	11.4 (13)	10.5 (25)	9.0 (33)	8.5 (44)	10.0 (161)
25-34 years old	3.3 (95)	--	2.9 (7)	2.7 (10)	6.2 (32)	2.9 (46)
≥35 years old	1.2 (34)	--	--	--	--	1.6 (25)
Missing	8.5 (240)	7.0 (8)	--	--	--	9.7 (156)
When you used to smoke, how often did you smoke?						
Less than weekly	6.8 (194)	24.6 (28)	13.0 (31)	7.6 (28)	7.0 (36)	4.4 (71)
Weekly (not every day)	11.2 (317)	24.6 (28)	8.8 (21)	13.9 (51)	12.6 (65)	9.5 (152)
Every day	75.2 (2134)	46.5 (53)	71.8 (171)	74.7 (275)	73.3 (379)	78.4 (1256)
Missing	6.8 (194)	4.3 (5)	6.3 (15)	3.8 (14)	7.2 (37)	7.7 (123)
When you used to smoke, how many cigarettes did you usually smoke in one day?						
1-10 cigarettes per day	39.7 (1128)	71.9 (82)	59.7 (142)	51.4 (189)	43.1 (223)	30.7 (492)
11-20 cigarettes per day	27.1 (768)	13.2 (15)	24.8 (59)	26.6 (98)	25.0 (129)	29.2 (467)
21-30 cigarettes per day	15.6 (443)	--	6.7 (16)	12.8 (47)	16.6 (86)	18.1 (290)
≥31 cigarettes per day	8.8 (250)	11.4 (13)	--	4.9 (18)	6.6 (34)	12.3 (197)
Missing or less than daily	8.8 (250)	--	--	4.3 (16)	8.7 (45)	9.7 (156)
How soon after waking did you usually have your first cigarette?						
5 minutes or less	13.8 (393)	39.5 (45)	16.4 (39)	13.9 (51)	14.7 (76)	11.4 (182)
6-30 minutes	23.3 (662)	12.3 (14)	17.6 (42)	22.3 (82)	24.4 (126)	24.8 (398)
31-60 minutes	26.7 (759)	22.8 (26)	31.1 (74)	31.3 (115)	28.0 (145)	24.9 (399)
61 or more minutes	14.1 (399)	11.4 (13)	16.0 (38)	15.2 (56)	10.4 (54)	14.9 (238)
Don’t smoke every day	13.0 (370)	7.9 (9)	11.8 (28)	12.5 (46)	14.7 (76)	13.2 (211)
Missing	9.0 (256)	6.1 (7)	7.1 (17)	4.9 (18)	7.7 (40)	10.9 (174)
Have you ever participated in any Quit Smoking Program, Service or Activity?						
No	94.2 (2673)	94.7 (108)	92.4 (220)	94.0 (346)	94.2 (487)	94.4 (1512)
Yes	5.8 (166)	5.3 (6)	7.6 (18)	6.0 (22)	5.8 (30)	5.6 (90)

^ Excludes participants with other gender (*N*=9) to protect confidentiality.
* Indicates a question where multiple responses are possible. This means that the column percentages may sum to more than 100.
-- indicates a cell that was suppressed due to small numbers.

Table S7: Recoding of smoking-related attitudinal outcomes

Original variable	Original coding	Binary recoding	Binary variable	Binary key
Do you agree that non-smokers miss out on gossip or yarning?	Not at all<br>A little bit<br>A fair bit/A lot<br>Missing	0=A little bit/a fair bit/a lot<br>1=Not at all<br>.=Missing	Non-smokers do not miss out on gossip or yarning	0=Disagree<br>1=Agree
Do you agree that your community disapproves of smoking?	Not at all<br>A little bit<br>A fair bit/A lot<br>Missing	0=Not at all<br>1=A little bit/a fair bit/a lot<br>.=Missing	My community disapproves of smoking	0=Disagree<br>1=Agree
Do you agree that smoking is not that risky?	Not at all<br>A little bit<br>A fair bit/A lot<br>Missing	0=A little bit/a fair bit/a lot<br>1=Not at all<br>.=Missing	Smoking is risky	0=Disagree<br>1=Agree
Do you think your smoking has made you sick?/ Do you think your past smoking will make you sick in the future?	No<br>Yes<br>Unsure<br>Missing	0=No<br>1=Yes<br>.=Unsure/ missing	Smoking has made me sick/Past smoking has made me sick	0=Disagree<br>1=Agree
Do you think your smoking will make you sick in the future?/ Do you think your past smoking will make you sick in the future?	Not at all<br>A little bit<br>A fair bit /A lot<br>Unsure<br>Missing	0=Not at all<br>1=A little bit/a fair bit/a lot<br>.=Unsure/missing	Smoking will make me sick in the future/ Past smoking will make me sick in the future	0=Disagree<br>1=Agree
Do you want to quit smoking? (current smokers only)	Not at all<br>A little bit<br>A fair bit /A lot<br>Unsure<br>Missing	0=Not at all<br>1=A little bit/a fair bit/a lot<br>.=Unsure/missing	Wants to quit smoking	0=No<br>1=Yes
Why do you want to quit?/What lead you to quit? (multi-selection)<br>• Advertising against smoking<br>• Medical advice<br>• My Health<br>• Health of my family<br>• Cost<br>• Pregnancy<br>• Too many non-smoking areas<br>• Pressure from family or friends<br>• Other<br>• Missing or indicated does not want to quit	Selected<br>Not selected	For each separate reason<br>0=Not selected<br>1=Selected	As original variable for each separate reason	0=Not selected<br>1=Selected
How long ago did you quit? (past smokers only)	1-5 months<br>6 months-1 year<br>1-2 years<br>2-5 years<br>>5 years<br>Missing	0=> 5 years<br>1=≤5 years<br>.=Missing	Length of time since quitting	0=>5years<br>1=≤5years
What helped you quit?	Selected<br>Not selected	For each separate support	As original variable for each separate support	0=Not selected

(multi-selection) (past smokers only)<br>• Smoking program<br>• Quitline<br>• Online support<br>• Health professional<br>• Family or friends<br>• Patches, gum, inhaler (NRT)<br>• Stop smoking medication<br>• Quit on my own<br>• Other<br>• Missing		0=Not selected<br>1=Selected		1=Selected

Table S8: Recoding of smoking-related behavioural outcomes

Original variable	Original coding	Binary recoding	Binary variable	Binary key
Smoking status	Never smoker<br>Current smoker<br>Past smoker<br>Missing	0=Past/Never smokers<br>1=Current smokers<br>.=Missing	Current smoker	0=No<br>1=Yes
Does anyone smoke in your home or in your car? (multi-selection)	No<br>Yes, inside the home<br>Yes, outside the home<br>Yes, in the car<br>Missing	For each separate question:<br>0=No<br>1=Yes<br>.=Missing	Anyone smokes in the home<br>Anyone smokes outside the home<br>Anyone smokes in the car	0=No<br>1=Yes
How old were you when you started smoking?	1-15 years<br>16-18 years<br>19-24 years<br>25-34 years<br>≥35 years<br>Missing	0=>16 years<br>1=≤15 years<br>.=Missing	Age commenced smoking ≤15 years	0=No<br>1=Yes
In the past year, have you tried to quit or reduce the amount you smoke? (multi-selection) (current smokers only)	Yes, tried to quit<br>Yes, tried to reduce<br>No<br>Missing	For each separate question:<br>0=No<br>1=Yes<br>.=Missing	Quit attempt in last 12 months<br>Reduction attempt in last 12 months	0=No<br>1=Yes
How often do you smoke?/ When you used to smoke, how often did you smoke?	Less than weekly<br>Weekly<br>Every day<br>Missing	0=Less than weekly/weekly<br>1= Everyday<br>.=Missing	Smokes daily/Smoked daily	0=No<br>1=Yes
How many cigarettes do you usually smoke in one day (CPD)?/ When you used to smoke, how many CPD?	1-10 CPD<br>11-20 CPD<br>21-30 CPD<br>≥31 CPD<br>Missing or <1	0=<21 CPD<br>1=≥21 CPD<br>.=Missing or <1	Smoking ≥21 cigarettes per day	0=No<br>1=Yes

How soon after waking do you usually have your first cigarette?/ How soon after waking did you usually have your first cigarette?	≤5 minutes<br>6-30 minutes<br>31-60 minutes<br>>60 minutes<br>Don’t smoke daily<br>Missing	0=>5 minutes<br>1=≤5 minutes	Time to first cigarette ≤5 minutes	0=No<br>1=Yes
Have you ever participated in any Quit Smoking Program, Service or Activity?	No<br>Yes	N/A	As original variable	0=No<br>1=Yes

Table S9: Associations between TIS exposure and smoking-related attitudes and behaviours in the total sample (includes current, past, and never smokers), adjusted for age, gender and remoteness

	PR adjusted for age, gender and remoteness (95%CI)
**Attitudes**	
Non-smokers do not miss out on gossip or yarning	
Non-TIS	1 (Ref)
TIS	1.01(0.98,1.04)
My community disapproves of smoking	
Non-TIS	1 (Ref)
TIS	1.02(<1.00,1.06)
Smoking is risky	
Non-TIS	1 (Ref)
TIS	1.01(0.97,1.06)
**Behaviours**	
Current smoker	
Non-TIS	1 (Ref)
TIS	0.99(0.92,1.06)
Anyone smokes in the home	
Non-TIS	1 (Ref)
TIS	0.84(0.73,0.96)
Anyone smokes outside the home	
Non-TIS	1 (Ref)
TIS	0.97(0.89,1.06)
Anyone smokes in the car	
Non-TIS	1 (Ref)
TIS	0.89(0.77,1.03)

Table S10. Associations between TIS exposure and smoking-related attitudes, among current and past smokers, adjusted for age, gender and remoteness

Current smoker	
	PR adjusted for age, gender and remoteness (95%CI)
Smoking has made me sick?	
Non-TIS	1 (Ref)
TIS	1.03(0.91,1.11)
Smoking will make me sick in the future	
Non-TIS	1 (Ref)
TIS	1.03(<1.00,1.06)
Wants to quit smoking	
Non-TIS	1 (Ref)
TIS	1.01(0.97,1.04)
Reasons for wanting to quit	
Advertising against smoking	
Non-TIS	1 (Ref)
TIS	0.66(0.40,1.09)
Medical advice	
Non-TIS	1 (Ref)
TIS	1.02(0.85,1.23)
My Health	
Non-TIS	1 (Ref)
TIS	1.02(0.94,1.10)
Health of my family	
Non-TIS	1 (Ref)
TIS	1.04(0.90,1.21)
Cost	
Non-TIS	1 (Ref)
TIS	1.01(0.92,1.10)
Pregnancy	
Non-TIS	1 (Ref)
TIS	1.54(0.62,3.85)
Too many non-smoking areas	
Non-TIS	1 (Ref)
TIS	0.98(0.66,1.45)
Pressure from family or friends	
Non-TIS	1 (Ref)
TIS	0.97(0.79,1.19)
**Past smoker**	
	PR adjusted for age, gender and remoteness (95%CI)
Past smoking has made you sick?	
Non-TIS	1 (Ref)
TIS	1.03(0.91,1.16)
Past smoking will make me sick in the future	
Non-TIS	1 (Ref)
TIS	1.04(0.95,1.14)
What led you to quitting	
Advertising against smoking	
Non-TIS	1 (Ref)
TIS	1.39(1.03,1.86)
Medical advice	
Non-TIS	1 (Ref)
TIS	0.96(0.81,1.15)
My Health	
Non-TIS	1 (Ref)
TIS	1.01(0.93,1.10)
Health of my family	
Non-TIS	1 (Ref)
TIS	0.98(0.83,1.15)
Cost	

Non-TIS	1 (Ref)
TIS	0.98(0.87,1.11)
Pregnancy	
Non-TIS	1 (Ref)
TIS	0.76(0.59,0.98)
Too many non-smoking areas	
Non-TIS	1 (Ref)
TIS	1.18(0.67,2.05)
Pressure from family or friends	
Non-TIS	1 (Ref)
TIS	1.23(>1.00,1.52)
What helped you quit?*	
Smoking program	
Non-TIS	1 (Ref)
TIS	0.77(0.49,1.22)
Quitline	
Non-TIS	1 (Ref)
TIS	0.51(0.26,<1.00)
Online support	
Non-TIS	1 (Ref)
TIS	0.58(0.11,2.91)
Health professional	
Non-TIS	1 (Ref)
TIS	0.70(0.48,1.02)
Family or friends	
Non-TIS	1 (Ref)
TIS	1.09(0.86,1.38)
Patches, gum, inhaler (NRT)	
Non-TIS	1 (Ref)
TIS	1.06(0.83,1.34)
Stop smoking medication	
Non-TIS	1 (Ref)
TIS	0.73(0.55,0.96)
Quit on my own	
Non-TIS	1 (Ref)
TIS	1.02(0.96,1.08)

Table S11: Associations between TIS exposure and smoking-related behaviours, among current and past smokers, adjusted for age, gender and remoteness

**Current smokers**	
	PR adjusted for age, gender and remoteness (95%CI)
Age commenced smoking ≤15 years	
Non-TIS	1 (Ref)
TIS	1.00(0.93,1.08)
Quit attempt in last 12 months	
Non-TIS	1 (Ref)
TIS	1.02(0.93,1.12)
Reduction attempt in last 12 months	
Non-TIS	1 (Ref)
TIS	1.01(0.93,1.11)
Smokes daily	
Non-TIS	1 (Ref)
TIS	1.04(>1.00,1.07)
Smoking ≥21 cigarettes per day	
Non-TIS	1 (Ref)
TIS	0.81(0.63,1.04)
Anyone smokes in the home	
Non-TIS	1 (Ref)
TIS	0.83(0.71,0.96)
Time to first cigarette ≤5 minutes	
Non-TIS	1 (Ref)
TIS	0.88(0.76,1.02)
Participation in any quit smoking program or activity	
Non-TIS	1 (Ref)
TIS	1.15(0.91,1.45)
**Past smokers**	
	PR adjusted for age, gender and remoteness (95%CI)
Age commenced smoking ≤15 years	
Non-TIS	1 (Ref)
TIS	0.95(0.88,1.02)
Smoked daily	
Non-TIS	1 (Ref)
TIS	0.99(0.96,1.02)
Past smoking ≥21 cigarettes per day	
Non-TIS	1 (Ref)
TIS	0.91(0.80,1.03)
Length of time since quitting <5years	
Non-TIS	1 (Ref)
TIS	1.05(0.95,1.17)
Anyone smokes in the home	
Non-TIS	1 (Ref)
TIS	1.05(0.71,1.55)
Past time to first cigarette <5 minutes	
Non-TIS	1 (Ref)
TIS	0.95(0.83,1.09)
Participation in any quit smoking program or activity	
Non-TIS	1 (Ref)
TIS	0.94(0.69,1.27)
